# Electrospun N‐Doped Carbon–Carbon Nanofibers with Enhanced Porosity for High‐Performance Zinc‐Ion Hybrid Supercapacitor Application

**DOI:** 10.1002/smsc.202400426

**Published:** 2025-02-25

**Authors:** Sayak Roy, Rajib Samanta, Sudip Barman

**Affiliations:** ^1^ School of Chemical Sciences National Institute of Science Education and Research (NISER) Bhubaneswar Odisha 752050 India; ^2^ Homi Bhabha National Institute (HBNI) 2nd floor, Training School Complex Anushaktinagar Mumbai 400094 India; ^3^ Centre for Interdisciplinary Sciences (CIS) NISER Jatni Odisha 752050 India

**Keywords:** electrospinning, N‐doped carbon, polyacrylonitrile, porous carbon nanofibers, zinc‐ion hybrid supercapacitors

## Abstract

Zinc‐ion hybrid supercapacitors (ZIHSCs) represent a promising frontier in high‐performance energy storage, offering greater energy density characteristic shown by batteries alongside high power yield and extended life of supercapacitors. Carbon materials, due to their inexpensiveness, abundance, and excellent conductivity, have been promising cathode choices for ZIHSCs. However, the application of electrospun carbon nanofibers as cathodes in ZIHSCs remains relatively unexplored. This study describes the synthesis of electrospun porous N‐doped carbon (NC)‐carbon nanofibers (NC‐CNFs) through a carbonization‐activation pathway. The NC‐CNFs achieves specific surface area (SSA) of 2426.6 m^2^g^−1^ and a specific capacity of 173.5 mAhg^−1^ at 0.1 Ag^−1^ in a ZIHSC setup. A maximum energy density (ED) and power density (PD) of 138.8 Wh kg^−1^ and 7998.9 W kg^−1^, respectively is also obtained. After 10 000 charge‐discharge cycles, the device retains 91.7% initial capacitance. Additionally, the charge storage performances of a symmetric supercapacitor (SC) and a ZIHSC, made of NC‐CNFs, are compared to prove the superiority of the ZIHSC over SC. This study highlights that incorporating NC into electrospun carbon nanofibers, with potassium hydroxide (KOH) activation, yields a ZIHSC cathode material with an optimal porous 1D morphology, large SSA, optimized nanofiber diameter, and efficient heteroatom doping, leading to excellent electrochemical performance.

## Graphical abstract

N‐doped carbon‐carbon nanofibers (NC‐CNFs) are synthesized via electrospinning followed by KOH activation. NC‐CNF^15^_800_ exhibits a high specific capacity of 173.5 mAh g^−1^ at 0.1 A g^−1^ in 2 M ZnSO_4_, achieving energy and power densities of 138.8 Wh kg^−1^ and 7998.9 W kg^−1^, respectively. N, O doping and porous morphology enhance electrochemical performance, enabling practical applications like illuminating a light emitting diode.

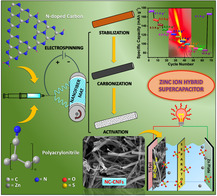

## 1 Introduction

The escalating environmental pollution and energy crisis from unrestricted fossil fuel use requires the development of clean and sustainable energy solutions.^[^ ^1^ ^]^ Supercapacitors, fuel cells, and batteries are examples of advanced energy storage technologies developed to improve energy consumption efficiency.^[^ ^2^, ^3^, ^4^ ^]^ While fuel cells and batteries have high energy density (ED) and efficiency in energy conversion, they are expensive and have low cyclic stability.^[^ ^5^ ^]^ On the other hand, outstanding power density (PD), exceptional cycling stability, and safety provided by aqueous electrolytes are the hallmarks of supercapacitors.^[^ ^6^ ^]^ Nevertheless, their poor ED and limited energy storage capacity continue to be serious disadvantages.^[^ ^7^ ^]^ To address these challenges, hybrid supercapacitors have emerged, combining the benefits of electrochemical double‐layer (EDL) capacitors and pseudocapacitors. Thus, they can achieve high ED due to Faradic reactions at anode and high PD by virtue of EDL capacitance (EDLC) at cathode.^[^ ^8^, ^9^ ^]^ Hybrid‐ion supercapacitors utilizing Li^+^, Na^+^, and K^+^ ions have proven to be extremely effective in renewable energy applications, providing rapid energy storage and extended lifespans, rendering them applicable to a wide spectrum of sectors ranging from portable electronic devices to automobile industry.^[^ ^10^, ^11^ ^]^ In spite of this, poor ED and safety concerns with highly reactive alkali metals still persist. Alternatively, multivalent ions such as Al^3+^, Ca^2+^, Mg^2+^, and Zn^2+^ provide quicker charge transfer kinetics, better capacitance, safety, and enhanced ED because they are able to deliver multielectron transfer in situations involving a single mole of ions. They are also found to be more affordable since they are abundant in nature. Aqueous zinc‐ion hybrid supercapacitors (ZIHSCs) featuring a battery‐type anode (Zn metal) and a capacitive cathode (carbon material) are particularly promising due to high theoretical specific capacity (820 mAh g^−1^) of zinc, less redox potential of −0.76 V (vs. SHE), high availability, low cost, better stability in electrochemical conditions, and low impact on the environment.^[^ ^12^, ^13^, ^14^, ^15^ ^]^ ZIHSCs combine the benefits of zinc‐ion batteries and electrochemical capacitors (ECs), where high electrical conductivity, large specific surface area (SSA), and variable pore size distribution (PSD) are necessary qualities for the cathode materials.^[^ ^16^, ^17^ ^]^ Currently, oxides based on manganese,^[^ ^18^ ^]^ vanadium,^[^ ^19^ ^]^ Prussian blue analogues,^[^ ^20^ ^]^ and some spinel oxides^[^ ^21^, ^22^ ^]^ along with carbon materials are used as cathodes for ZIHSCs. While others suffer from poor structural and cyclic stability, carbon‐based materials, with their tunable surface area and enhanced stability, offer significant electrochemical advantages.^[^ ^23^ ^]^ The cathodic reaction comprises of the fast adsorption/desorption of electrolyte ions and the anodic reaction involves the reversible plating/deplating of Zn^2+^ to ensure high PD and ED values for ZIHSCs, respectively.^[^ ^24^, ^25^ ^]^ Many porous carbon compounds have recently been researched for use as ZIHSC cathode materials. For instance, An et al.^[^ ^7^ ^]^ assembled a Zn||NC‐CMPAs ZIHSC, which exhibited a specific capacity of 195 mAh g^−1^ under 0.1 A g^−1^, with an ED of 157.47 Wh kg^−1^ and a PD of 8020.90 W kg^−1^. Also, Wang et al.^[^ ^26^ ^]^ developed hierarchical porous carbon functionalized with oxygen‐containing groups demonstrating a specific capacity of 169.4 mAh g^−1^ under 0.1 A g^−1^. Studies have established that the bidirectional SO_4_^2−^ adsorption/desorption on the porous cathode improves rate capability and cyclic durability.^[^ ^16^ ^]^ Despite the potential of carbon materials like graphene, carbon nanotubes (CNTs), biomass‐derived porous carbons, and activated carbons (ACs), their inadequate porosity and irregular frameworks limit their electrochemical performance, which results in significant ion diffusion resistance.^[^ ^14^ ^]^ Moreover, the synthesis procedures for graphene and CNTs are often expensive, complicated, and environmentally hazardous.^[^ ^17^ ^]^ On the other hand, metal oxide‐based materials such as MnO_2_ nanorods//AC showed high power characteristics (3.3–13.0 kW kg^−1^) due to their rapid charge/discharge rate (2–17 s) but very low specific capacity (54.1 mAh g^−1^) and ED (34.8 Wh kg^−1^).^[^ ^27^ ^]^ Other state‐of‐the art cathode materials for ZIHSCs, except carbon and metal oxides, include MXenes and redox‐active polymers.^[^ ^28^ ^]^ Wang et al. synthesized a porous 3D MXene (Ti_3_C_2_T_*x*_)–reduced graphene oxide aerogel cathode with a high specific capacitance (C_s_) of 128.6 F g^−1^ at 0.4 A g^−1^ and a high ED of 34.9 Wh kg^−1^ at a PD of 279.9 W kg^−1^. The material showed a 95% capacitance retention for 75 000 cycles at 5 Ag^−1.^^[^ ^29^ ^]^ A free‐standing redox‐active polydopamine (PDA)/carbon cloth‐based cathode was prepared by Huang et al. with an areal capacity of 1.25 mAh cm^−2^ and a capacity retention of 100% over 10 000 cycles at 10 mA cm^−2.^^[^ ^30^ ^]^

In such a scenario, one intriguing, yet untested, option for ZIHSC cathode materials is electrospun carbon nanofibers (CNFs). Nevertheless, carbon fibers lack the SSA and pore volume of AC, limiting their EDLC.^[^ ^31^ ^]^ Zinc ion and other anion adsorption is also hampered by the intrinsic hydrophobicity of carbon fibers, which increases electrode/electrolyte interfacial resistance. In order to increase the chemisorption of metal ions on carbon electrodes by enhancing their hydrophilicity, surface modification has been found to be an efficient technique.^[^ ^32^, ^33^ ^]^ Also, it is a common practice to modify carbon fibers by incorporating appropriate percentages of heteroatoms, such as N and O, which greatly increase their surface area and hydrophilicity.^[^ ^16^ ^]^

Here, we report the electrospun synthesis of porous CNFs integrated with NC for high‐performance ZIHSCs. The final product (NC‐CNFs) was synthesized through carbonization at elevated temperature succeeding activation by KOH. The optimized sample, having an ideal amount of NC and activated at an optimal temperature, attained a maximum C_s_ of 418 F g^−1^ under 1 A g^−1^ in 1 M H_2_SO_4_, demonstrating excellent cyclic durability of 92.1% over 10 k cycles. Additionally, a symmetric supercapacitor device (NC‐CNF^15^_800_||NC‐CNF^15^_800_) was assembled, which exhibited a C_s_ of 245.7 F g^−1^ under 1 A g^−1^ and 88.6% cyclic stability after 10 k charge–discharge cycles, showing superior ED and PD. Most interestingly, the material showed an extraordinary specific capacity of 173.5 mAh g^−1^ under 0.1 A g^−1^ in a ZIHSC configuration (Zn||NC‐CNF^15^_800_). The ZIHSC device reached the highest PD and ED of 7998.9 W kg^−1^ and 138.8 Wh kg^−1^, respectively. Due to its exceptional 1D morphology, large SSA, and optimum N, O doping within the CNFs, NC‐CNF^15^_800_ exhibits excellent energy storage performance.

## 2 Experimental Section

### 2.1 Synthesis of N‐Doped Carbon–Carbon Nanofibers (NC‐CNFs)

A microwave‐assisted method was used to synthesize NC from formamide (HCONH_2_), using a previously reported procedure.^[^ ^34^ ^]^ For the synthesis of NC‐CNFs, N,N‐dimethylformamide (DMF) (17.5 mL) was used as a solvent to solubilize the polymer polyacrylonitrile (PAN) (1 g) under continuous stirring at 600 rpm and heating at 60 °C. Subsequently, various amounts of NC (0, 0.1, 0.15, and 0.3 g) were dispersed in the mixture and stirred at 1200 rpm for 12 h. After that, the resultant solution was fed into two syringes of volume 10 mL each and electrospinning was performed (Espin Nano; Pico. India), under a voltage difference of 20 kV and a feeding rate of 1 mL hr^−1^. The needle tip and the aluminum foil drum collector were separated by a distance of 15 cm, with the drum rotating at 400 rpm.

**Scheme 1:**
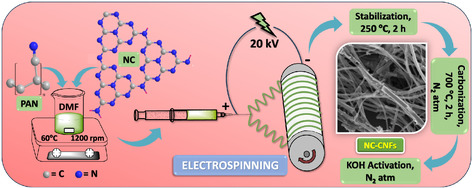
Scheme illustrating the synthesis of NC‐CNFs.

After electrospinning, stabilization of the nanofiber mat was performed at 250 °C for 2 h (5 °C min^−1^) in an air furnace. This was followed by carbonization for 2 h at 700 °C (5 °C min^−1^) in a N_2_ atmosphere. The carbonized product was subsequently activated for 1 h (5 °C min^−1^), also in a similar atmosphere at 700, 800, and 900 °C using KOH with a ratio of 2:1 between KOH and carbonized nanofibers. The formed products were meticulously washed with deionized water and hydrochloric acid (HCl) until the excess KOH was neutralized, followed by overnight drying at 120 °C, yielding the NC‐CNF^*x*^_*y*_ samples. Here, “*x*” denotes the NC doping amount while “*y*” represents the activation temperature, that is, NC‐CNF^00^_800_ (0 g NC; 800 °C), NC‐CNF^10^_800_ (0.1 g NC; 800 °C), NC‐CNF^15^_800_ (0.15 g NC; 800 °C), NC‐CNF^30^_800_ (0.3 g NC; 800 °C), NC‐CNF^15^_700_ (0.15 g NC; 700 °C), and NC‐CNF^15^_900_ (0.15 g NC; 900 °C). The synthesis procedure of the NC‐CNFs is detailed in **Scheme** **1**. In the present work, two types of control over the nanofiber morphology have been adopted to obtain the optimized NC‐CNF, that is, optimization of 1) the amount of NC incorporated into the nanofibers and 2) the activation temperature of the nanofibers. The resulting material is expected to exhibit ideal heteroatom (N, O) doping, surface porosity, and nanofiber thickness, the synergistic effect of which will improve its performance as a ZIHSC cathode material.

## 3 Results and Discussion

### 3.1 Structural and Morphological Characterizations

**Figure 1:**
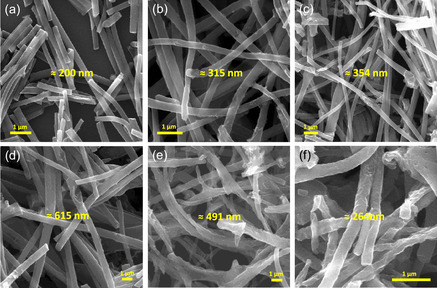
SEM images of a) NC‐CNF^00^_800_, b) NC‐CNF^10^_800_, c) NC‐CNF^30^_800_, d) NC‐CNF^15^_700_, e) NC‐CNF^15^_800_, and f) NC‐CNF^15^_900_ nanofibers, along with their average diameters.

The structural characteristics of NC‐CNF^*x*^_*y*_ were assessed using powder X‐ray diffraction analysis, displayed in Figure S1 (Supporting Information). At the 2*θ* of 25.9° and 43.4°, two peaks occurred which can be attributed to the two crystal planes of graphitic carbon, that is, (002) and (100), respectively. The broad diffraction peaks indicate that the carbon is amorphous in nature and has a lower crystallinity. Scanning electron microscopy (SEM) was utilized to inspect the morphological characteristics of NC‐CNF^*x*^_*y*_. A comparison of the morphologies of NC‐CNF^00^_800_, NC‐CNF^10^_800_, NC‐CNF^30^_800_, NC‐CNF^15^_700_, NC‐CNF^15^_800_, and NC‐CNF^15^_900_ is shown in **Figure** **1**a–f, respectively. The nanofibers without NC (NC‐CNF^00^_800_) had a mean diameter of about 200 nm (Figure 1a). Introducing NC initially increases the mean diameter from 315 nm in NC‐CNF^10^_800_ (Figure 1b) to 491 nm in NC‐CNF^15^_800_ (Figure 1e) and then decreases to 354 nm in NC‐CNF^30^_800_ (Figure 1c). Additionally, surface porosity and defects increase progressively from NC‐CNF^15^_700_ to NC‐CNF^15^_900_, with mean diameters shrinking from 615 to 264 nm. NC‐CNF^15^_700_ (Figure 1d) shows relatively smoother nanofiber surface, while the porosity increases in NC‐CNF^15^_800_ (Figure 1e) ultimately leading to irregularly shaped nanofibers in NC‐CNF^15^_900_ (Figure 1f). These observations suggest that higher activation temperatures enhance surface defects. NC‐CNF^15^_800_ exhibits ideal nanofiber thickness and porosity, which optimize its capacity for charge storage.

**Figure 2:**
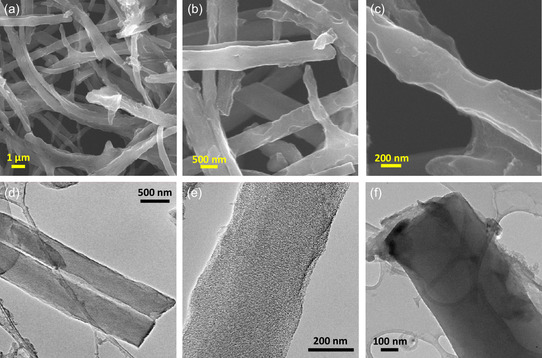
a–c) FESEM images of NC‐CNF^15^_800_ from lower magnification to higher and d–f) TEM images of NC‐CNF^15^_800_ on a lacey carbon grid.

The morphology of NC‐CNF^15^_800_ is shown at different magnifications in **Figure** **2**a–c. At lower magnification, the Field emission SEM (FESEM) image confirms the formation of a nanofiber network (Figure 2a). Also, Figure 2b,c provides the magnified view of the porous and rough surfaces of the nanofibers, highlighting deformities caused by KOH activation. The activation process involves a set of reactions, starting with reaction between the carbon content and KOH, during which K_2_CO_3_ is formed at ≈400 °C.

(1) $$2\text{ C}+6\text{ KOH }\to 2{\text{ K}}_{2}{\text{CO}}_{3}+\;2\text{ K}+\;3{\text{ H}}_{2}$$

When the temperature rises beyond 600 °C, K_2_CO_3_ breaks down into K_2_O and volatilizes out as CO_2_, which increases the porosity and surface defects of the nanofibers.

(2) $$K_{2}{\text{CO}}_{3}\;\to K_{2}O+{\text{CO}}_{2}$$

Meanwhile, K_2_CO_3_ also reacts with carbon, producing metallic K and generating porosity by oxidizing carbon into CO.

(3) $$2\text{ C}+K_{2}{\text{CO}}_{3}\;\to 2\text{ K}+\;3\text{ CO}$$

The as‐prepared metallic K intercalates into the carbon matrix, expanding the carbon layers. Activation at high temperatures volatilizes most of the K, leaving behind exfoliated carbon structures with high porosity and large surface areas. Residual K is removed by washing with HCl and water postactivation.^[^ ^35^, ^36^, ^37^ ^]^ Consequently, at a high magnification, the uneven nanofiber surface can be seen prominently in Figure 2c. Additionally, the porous NC‐CNFs were further examined by transmission electron microscopy (TEM). Figure 2d–f and Figure S2 (Supporting Information) depict the low‐ and high‐resolution TEM (HRTEM) images of NC‐CNF^15^_800_, respectively. While Figure 2d shows the uneven nanofiber surface and edges, Figure 2e displays the high porosity and disorderness of a single NC‐CNF^15^_800_ nanofiber and Figure 2f displays the usual carbon boundaries and surface edge defects. The growth of microporous structures (marked by green circles) is shown by recurring dark and light patterns at the nanofiber edges as perceived from the HRTEM image (Figure S2, Supporting Information). By creating defects on the NC‐CNF^15^_800_ sample during synthesis, KOH activation helps to improve the porous structure of the sample.^[^ ^38^ ^]^ The conductivity and surface wettability of the material are greatly enhanced by this porosity, which makes NC‐CNF materials ideal for energy storage applications like ZIHSCs. Table S1 (Supporting Information) provides the mass percentages of C, N, and O for the NC‐CNF^*x*^_*y*_ samples as determined from SEM–energy‐dispersive X‐ray spectroscopy (EDX). It is found that the nitrogen content (% N) rises with increasing NC doping; however, it drops with increasing activation temperature, attaining an optimum 7.60% N in NC‐CNF^15^_800_. The activation process also increases the oxygen content (% O) due to the creation of functionalities on the surface such as C=O, C—O, C—OH, etc. enhancing hydrophilicity and creating surface defects. The elemental mapping images of NC‐CNF^15^_800_ have been displayed in Figure S3 (Supporting Information) where the arrangement of C, N, and O elements throughout the sample is clearly observed.

**Figure 3:**
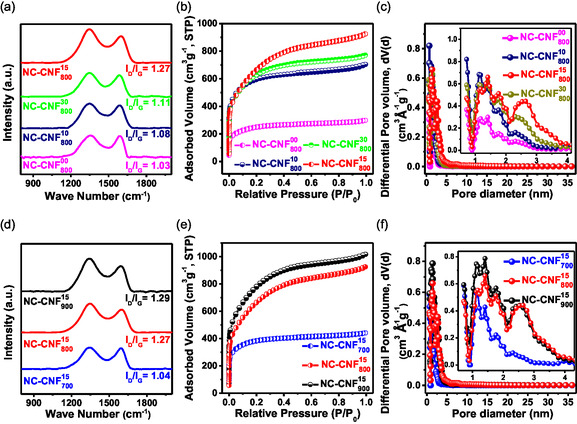
a,d) Raman spectra, b,e) N_2_ adsorption plot, and c,f) PSD of NC‐CNF^*x*^_*y*_.

Using Raman spectroscopy, a comparison between the degree of graphitization and disorderness in NC‐CNF^*x*^_*y*_ was estimated (**Figure** **3**a,d). Two peaks, indicating the D and G bands, were seen in each sample at ≈1345 and 1590 cm^−1^, respectively. The G band denotes the graphitic sp^2^ carbon content, whereas the D band indicates structurally disordered sp^3^ carbon present in the matrix. An increase in the D band intensity suggests greater disorder in the carbon structure. The degree of disorder in a material is indicated by the ratio of intensities of the D and G bands (*I*_D_/*I*_G_). The introduction of heteroatoms into the carbon matrix and activation at elevated temperatures intensifies the D band. The value of *I*_D_/*I*_G_ increased from 1.03 for NC‐CNF^00^_800_ to 1.27 for NC‐CNF^15^_800_ (Figure 3a) and from 1.04 for NC‐CNF^15^_700_ to 1.29 for NC‐CNF^15^_900_ (Figure 3d), indicating increased disorder with higher NC doping and higher activation temperature, thereby enhancing the capacity of energy storage.^[^ ^39^ ^]^ In order to investigate the porosity parameters of NC‐CNF^*x*^_*y*_, Brunauer–Emmett–Teller (BET) measurements were used. This is important because materials with robust pore networks have enhanced electrode–electrolyte interaction. The N_2_ sorption isotherms for each sample are displayed in Figure 3b,e. These isotherms exhibit the typical type‐I isotherm (microporous), in which adsorbed N_2_ volume rises sharply at a lower relative pressure range.^[^ ^40^ ^]^ Detailed parameters are recorded in **Table** **1**a,b. The micropore surface area and SSA were determined using t‐plot and BET methods, respectively. The total pore volumes (*V*_t_) of NC‐CNF^00^_800_, NC‐CNF^10^_800_, NC‐CNF^15^_800_, and NC‐CNF^30^_800_ were calculated to be 0.46, 1.09, 1.43, and 1.19 cc g^−1^, respectively, while their SSAs were 887.4, 2165.2, 2426.6, and 2265.3 m^2^ g^−1^, respectively (Table 1a). Thus, as the amount of NC doping increases, the SSA also increases from NC‐CNF^00^_800_ up to NC‐CNF^15^_800_, and again decreases in NC‐CNF^30^_800_ as the limit of NC incorporation was reached. On the other hand, NC‐CNF^15^_700_ and NC‐CNF^15^_900_ have SSAs of 1400.2 and 2811.7 m^2^ g^−1^, respectively, along with *V*_t_s of 0.68 and 1.57 cc g^−1^, respectively (Table 1b). This can be explained by the fact that with increasing activation temperature more amount of carbon content volatilizes out, leaving behind greater porous materials with larger SSAs. Thus, activation temperature and NC doping have a critical contribution in the creation of porous CNFs. For the optimized nanofiber sample, NC‐CNF^15^_800_, the S_micro_/S_BET_ was found to be 52.8%, least among all other control samples. This finding suggests that in NC‐CNF^15^_800_ the fraction of mesoporous and external surface area was greater as compared to other samples. It is established that mesoporous materials have fast electrolyte ion transport onto the inner electrode surface, since the mesopores operate as electrolyte channels or reservoirs, resulting in increased C_s_ at rapid charge–discharge rates.^[^ ^41^ ^]^ The nonlocal density functional theory model was implemented to perform the PSD analysis for NC‐CNF^*x*^_*y*_, which is illustrated in Figure 3c,f. It is observed that the pore network of the samples consists of a mix of mesopores and micropores, which is extremely advantageous since aqueous electrolyte ions can easily circulate through this porous structure, due to which the capacitance values are increased.^[^ ^42^ ^]^ Additionally, the *V*_t_ of the samples increases with the SSA. The better ion transport and adsorption are facilitated by high SSA of NC‐CNF^15^_800_, optimum ratio of micropores and mesopores, and adequate N and O doping.

**Table 1:** Detailed surface properties and pore analysis of NC‐CNF^*x*^_*y*_.

Material	BET surface area (S_BET_) [m^2^ g^−1^]	Micropore surface area (S_micro_) [m^2^ g^−1^]	Total pore volume (*V*_t_) [cc g^−1^]	Micropore volume (*V*_micro_) [cc g^−1^]	Average pore diameter (*D*_a_) [nm]	Percentage [%] of S_micro_/S_BET_
Table 1 (a)						
NC‐CNF^00^_800_	887.4	739.4	0.46	0.32	1.16	83.3
NC‐CNF^10^_800_	2165.2	1859.5	1.09	0.79	1.15	85.8
**NC‐CNF** ^**15**^_**800**_	**2426.6**	**1282.2**	**1.43**	**0.56**	**1.22**	**52.8**
NC‐CNF^30^_800_	2265.3	1675.0	1.19	0.73	1.13	73.9
Table 1 (b)						
NC‐CNF^15^_700_	1400.2	1223.2	0.68	0.52	1.08	87.3
**NC‐CNF** ^**15**^_**800**_	**2426.6**	**1282.2**	**1.43**	**0.56**	**1.22**	**52.8**
NC‐CNF^15^_900_	2811.7	1743.7	1.57	0.59	1.18	62.0

According to our study, incorporating NC into CNFs led to changes in properties such as thickness, disorderness, surface area, and porosity. The properties were improved up to a specific amount of NC, beyond which further addition negatively impacted the properties since NC fails to get incorporated into the nanofibers (Figure S4, Supporting Information). For instance, increasing NC from 0.1 g (NC‐CNF^10^_800_) to 0.15 g (NC‐CNF^15^_800_) enhanced these characteristics, but further doping up to 0.3 g (NC‐CNF^30^_800_) led to a decline. Therefore, 0.15 g is the optimal NC doping concentration for CNFs. Again, an increase in activation temperature from 700 °C (NC‐CNF^15^_700_) to 900 °C (NC‐CNF^15^_900_) resulted in the improvement of all the above properties but also caused a decrease in the percent N doping. In this case, 800 °C was found to be the activation temperature that adequately balanced N doping and surface porosity, thus making NC‐CNF^15^_800_ the optimized nanofiber sample, which is expected to have better electrochemical performance than the other control samples.

**Figure 4:**
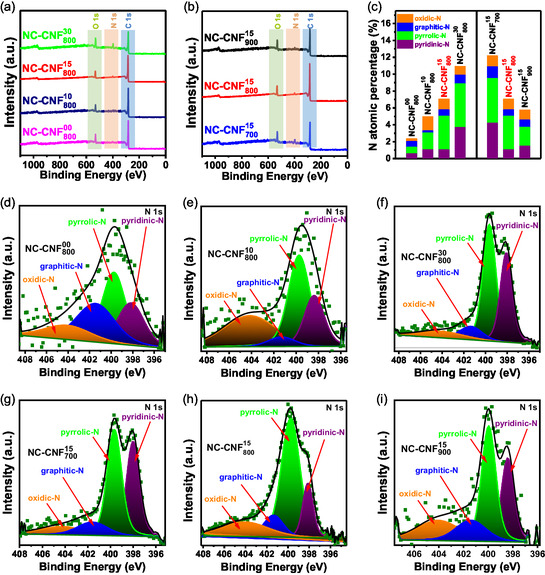
a,b) XPS survey spectra; c) atomic contents of different N species in NC‐CNF^*x*^_*y*_. N 1s spectra of d) NC‐CNF^00^_800_, e) NC‐CNF^10^_800_, f) NC‐CNF^30^_800_, g) NC‐CNF^15^_700_, h) NC‐CNF^15^_800_, and i) NC‐CNF^15^_900_.

X‐ray photoelectron spectroscopy (XPS) was used to examine the chemical states and surface elemental compositions of NC‐CNFs. All the samples showed peaks corresponding to C 1s, N 1s, and O 1s in the XPS survey spectra (**Figure** **4**a,b). The lack of unwanted peaks verified the absence of contaminants. The atomic percentages of the elements on the sample surfaces are listed in **Table** **2**. The nitrogen content varied between 4.94% and 12.15% for NC‐CNF^10^_800_ to NC‐CNF^30^_800_ and NC‐CNF^15^_700_ to NC‐CNF^15^_900_, significantly higher than in NC‐CNF^00^_800_ (2.31%). This suggests that the NC has a prominent role in the higher level of N‐doping in all the samples other than NC‐CNF^00^_800_. Furthermore, an increasing C:N ratio from NC‐CNF^15^_700_ to NC‐CNF^15^_900_ indicates a denitrification reaction occurring with increasing activation temperature. The peaks were deconvoluted using high‐resolution scans, which allowed distinguishing between different bonding states of the elements. Four constituent peaks were identified from the deconvolution of the C 1s spectra in all the NC‐CNFs, with corresponding binding energies of ≈284.6, ≈286, ≈287.6, and ≈288.9 eV (Figure S5a–f, Supporting Information), representing C=C/C—C, C—N/C—O, C=O/C=N, and O=C—O, respectively.^[^ ^43^, ^44^ ^]^ The existence of pyridinic N, pyrrolic N, graphitic N, and oxidic N in the NC‐CNFs was confirmed by the high‐resolution spectra of N 1s (Figure 4d–i), which can be fitted into the corresponding peaks at ≈ 398.1, ≈ 399.8, ≈ 401.4, and ≈ 404 eV, respectively.^[^ ^44^, ^45^, ^46^ ^]^ The percentage compositions of these N‐containing species are mentioned in Table 2 and graphically represented in Figure 4c. Here, it is observed that the % N increases from NC‐CNF^00^_800_ to NC‐CNF^30^_800_ and decreases from NC‐CNF^15^_700_ to NC‐CNF^15^_900_, which is consistent with the SEM–EDX results. Among all the samples, NC‐CNF^15^_800_ shows the perfect optimization of N content and porosity characteristics. About 71% of the nitrogen species in NC‐CNF^15^_800_ are pyridinic and pyrrolic Ns, which are critical for pseudocapacitance, providing additional active sites to enhance overall capacitance. These species also improve Zn^2^^+^ ion chemisorption and hydrophilicity of the electrode, which facilitate Zn^2^^+^ storage in the ZIHSC device.^[^ ^47^ ^]^ In addition, graphitic N enhances electron transport, improving the conductivity of the carbon matrix.^[^ ^48^ ^]^ Finally, the presence of oxygen‐containing functional groups, that is, C=O, C—O, and C—OH in all the NC‐CNF^*x*^_*y*_ samples, was confirmed by the deconvoluted O 1s spectra, which fits accurately into corresponding three peaks at ≈530.9, ≈532.5, and ≈535.6 eV, respectively^[^ ^49^ ^]^ (Figure S6a–f, Supporting Information). Additionally, increased nitrogen doping from NC‐CNF^10^_800_ to NC‐CNF^30^_800_ also enhanced the hydrophilicity of the material, as evident by a reduction in their water contact angle from 53.3° to 33.9° (Figure S7, Supporting Information).

**Table 2:** Elemental atomic percentages and percentage compositions of different N species of NC‐CNF^*x*^_*y*_.

Sample Name	Atomic percentage [%]			Percentage composition of N species [%]			
	C	N	O	Pyridinic N	Pyrrolic N	Graphitic N	Oxidic N
Table 2 (a)							
NC‐CNF^00^_800_	79.32	2.31	18.36	22.67	33.65	29.83	13.84
NC‐CNF^10^_800_	74.78	4.94	20.27	20.92	40.24	5.39	33.44
**NC‐CNF** ^**15**^_**800**_	**71.28**	**7.13**	**21.59**	**14.63**	**56.59**	**11.07**	**17.71**
NC‐CNF^30^_800_	66.39	10.90	22.68	33.64	47.48	9.52	9.36
Table 2 (b)							
NC‐CNF^15^_700_	71.13	12.15	16.72	34.42	43.57	11.46	10.54
**NC‐CNF** ^**15**^_**800**_	**71.28**	**7.13**	**21.59**	**14.63**	**56.59**	**11.07**	**17.71**
NC‐CNF^15^_900_	70.83	5.72	23.45	25.42	38.93	15.13	20.51

## 4 Electrochemical Analysis

### 4.1 Electrochemical Behavior in Three‐Electrode System

**Figure 5:**
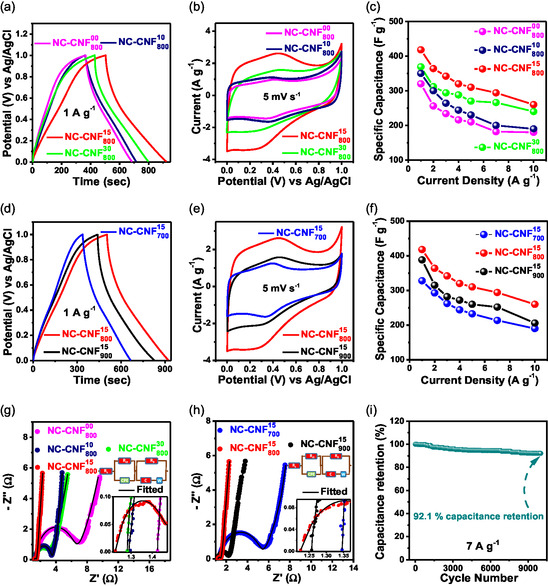
a,d) GCD curves under 1 A g^−1^, b,e) CV curves at 5 mV s^−1^ c,f) C_s_ versus current density, g,h) Nyquist plot for NC‐CNF^*x*^_*y*_, and i) % capacitance retention versus number of cycles for NC‐CNF^15^_800_ at 7 A g^−1^ in 1M H_2_SO_4_ electrolyte.

A three‐electrode setup was utilized to evaluate the charge storage properties of NC‐CNF^*x*^_*y*_ samples in a 1 M H_2_SO_4_ medium. **Figure** **5**a presents the comparative galvanostatic charging–discharging (GCD) curves for NC‐CNF^00^_800_, NC‐CNF^10^_800_, NC‐CNF^15^_800_, and NC‐CNF^30^_800_ at 1 A g^−1^ whereas Figure 5d displays those for NC‐CNF^15^_700_, NC‐CNF^15^_800_, and NC‐CNF^15^_900_ in between a voltage range of 0 and 1 V. NC‐CNF^15^_800_ had the longest discharge duration, which suggests that it has a higher C_s_ value than the other samples. For NC‐CNF^00^_800_, NC‐CNF^10^_800_, NC‐CNF^15^_800_, and NC‐CNF^30^_800_, the C_s_ values were determined to be 320, 350, 418, and 369 F g^−1^, respectively, and for NC‐CNF^15^_700_ and NC‐CNF^15^_900_, the C_s_ values were 328 and 388 F g^−1^, respectively at 1 A g^−1^. Figure 5b,e displays the comparative cyclic voltammetry (CV) plots at 5 mV s^−1^. Each CV curve reveals a semirectangular form with a couple of oxidation–reduction peaks, suggesting that the pseudocapacitance in NC‐CNFs also contributes to the charge storage, in addition to EDLC. The O and N functionalities on the surface allow redox reactions to occur which cause pseudocapacitance, whereas the EDLC behavior is attributed to the porous carbon matrix. Compared to other samples with varying NC ratios and activation temperatures, NC‐CNF^15^_800_ displayed a considerably greater current density at 5 mV s^−1^, suggesting its superior charge‐storing capabilities, consistent with the GCD results. This implies that the highest electrochemical performance is displayed by the sample with 0.15 g of NC and activated at 800 °C (NC‐CNF^15^_800_). The GCD and CV curves of all the NC‐CNF^*x*^_*y*_ samples have been also presented in same figures (Figure S8a,b, Supporting Information), for better understanding. The GCD profiles at varying current densities and the CV curves at different sweep rates for NC‐CNF^00^_800_, NC‐CNF^10^_800_, NC‐CNF^30^_800_, NC‐CNF^15^_700_, NC‐CNF^15^_800_, and NC‐CNF^15^_900_ are shown in Figure S9–S14 (Supporting Information), respectively. The C_s_ values for NC‐CNF^15^_800_ (calculated using Equation (S1), Supporting Information) at current densities of 1, 2, 3, 4, 5, 7, and 10 A g^−1^ are 418, 364, 342, 320, 310, 294, and 260 F g^−1^, respectively. All GCD profiles show roughly triangular curves, which denote good cycle performance and rate capability. Nevertheless, the curves are not fully symmetrical due to the N and O doping. The area under the CV curves increases for each sample when the sweep rate is increased since they achieve larger current densities. The C_s_ value witnesses a drop with increasing current, which is true for all the samples, as observed in Figure 5c,f. Figure 5g,h show the Nyquist spectra for NC‐CNF^*x*^_*y*_ samples. The high‐frequency zone, characterized by a semicircle, denotes the charge transfer resistance (*R*_ct_) between the electrolyte and electrode, whereas the solution resistance (*R*_s_) is found at the point where the spectrum merges with the real axis. Smaller the semicircle, smaller is the *R*_ct_ and greater is the pseudocapacitive contribution in charge storage.^[^ ^50^ ^]^ In contrast to others, NC‐CNF^15^_800_ has a smaller semicircle, indicating better charge storage properties. Table S2 (Supporting Information) contains the R_s_ and R_ct_ values for each sample, determined by the Nyquist plot circuit fitting. The material also exhibited cyclic stability for 10 k cycles maintaining 92.1% of its capacitance (Figure 5i).

The following elements are accountable for the exceptional electrochemical performance of NC‐CNF^15^_800_. 1) An enormous surface is provided by the exterior of the nanofibers due to their unique 1D structures. 2) The high porosity of the nanofibers and the presence of an abundant number of hydrophilic (N, O) surface functionalities promote better electrolyte diffusion. Furthermore, the well‐developed pore network improves wettability, increasing the electrolyte/electrode interaction which in turn boosts the charge storage capability. 3) The presence of functional groups, containing N and O, causes Faradic redox processes, which contribute to pseudocapacitance. This heteroatom doping improves the charge storage properties of the material.

### 4.2 Electrochemical Performance of NC‐CNF^15^_800_ Symmetric Supercapacitor (NC‐CNF^15^_800_||NC‐CNF^15^_800_)

**Figure 6:**
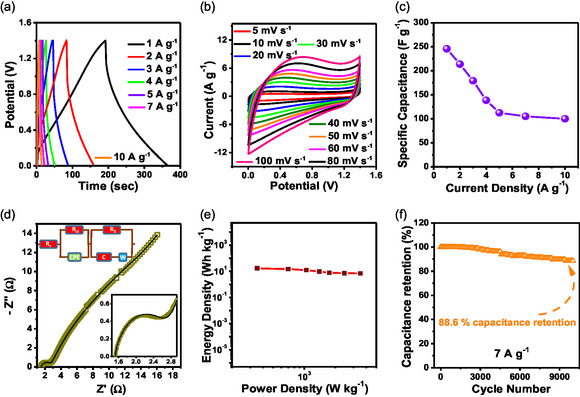
a,b) GCD and CV plot profiles of a NC‐CNF^15^_800_||NC‐CNF^15^_800_ device. c) C_s_ versus current densities plot. d) Nyquist plot. e) Ragone plot. f) Cycling performance for 10 000 cycles in 1 M H_2_SO_4_ electrolyte.

A symmetric SC device, NC‐CNF^15^_800_||NC‐CNF^15^_800_, was set up in a two‐electrode configuration, with NC‐CNF^15^_800_ both as anode and cathode and 1 M H_2_SO_4_ electrolyte. Figure S15 (Supporting Information) shows the CV curves at increasing voltages varying from 0–1 V to 1.6 V. The potential of the device is limited to 0–1.4 V for all electrochemical characterizations, beyond which a steep rise in current in the CV curve signifies H_2_O decomposition in aqueous medium. **Figure** **6**a displays the reversible charge/discharge performance as demonstrated by the roughly symmetric triangular GCD curves at various current densities. The N and O atoms present in the NC‐CNFs cause slight variation in the triangular GCD curves, leading to enhanced activity of the device. The CV curves of the device were obtained under increasing scan rates (Figure 6b). C_s_ values of the device (from Equation (S2), Supporting Information) were 245.71, 213.71, 179.14, 138.29, 112.36, 104.9, and 99.86 F g^−1^ for current densities of 1, 2, 3, 4, 5, 7, and 10 A g^−1^, respectively, as shown in Figure 6c. The equivalent circuit for the electrochemical impedance spectrum (EIS) can be seen in the inset of the EIS plot (Figure 6d), which reveals *R*_s_ and *R*_ct_ values to be 1.51 and 1.19 Ω, respectively. The ED and PD values of NC‐CNF^15^_800_||NC‐CNF^15^_800_ are additional metrics to evaluate its potential for practical use. The ED versus PD (Ragone) plot has been illustrated in Figure 6e, where the values were calculated using Equation (S3) and (S4). The device reached the highest PD and ED values of 3500.1 W kg^−1^ and 16.72 Wh kg^−1^, respectively. According to the cyclic stability plot (Figure 6f), the device retained 88.6% capacitance at 7 A g^−1^ beyond 10 k cycles.

In order to understand the electrochemical kinetics of the device, Dunn's method was utilized to estimate the diffusive and capacitive contributions with the following formula.

(4) $$i\left(V\right)=\;k_{1}v+\;k_{2}v^{1/2}$$

where, *i(V)* is the current density, *v* is the sweep rate, and *k*_1_ and *k*_2_ are potential‐dependent constants. The capacitive (*k*_1_ *v*) and diffusion‐limited (*k*_2_ *v* ^1/2^) contributions can be derived for different scan rates from the *i* versus *v* ^1/2^ graph.^[^ ^51^ ^]^ As shown in Figure S16a (Supporting Information), the plot of capacitive contribution at 5 mV s^−1^ was obtained using Equation (4). The percentage of capacitive charge storage was calculated to be 62.21, 69.67, 77.87, 83.62, 88.70, and 93.83% at 5, 10, 20, 30, 40, and 50 mV s^−1^, respectively. The charge storage process is majorly capacitive controlled, as seen in the corresponding plot of diffusive versus capacitive contributions at various scan rates in Figure S16b (Supporting Information).

### 4.3 Electrochemical Performance of the NC‐CNF^15^_800_ ZIHSC (Zn||NC‐CNF^15^_800_)

**Figure 7:**
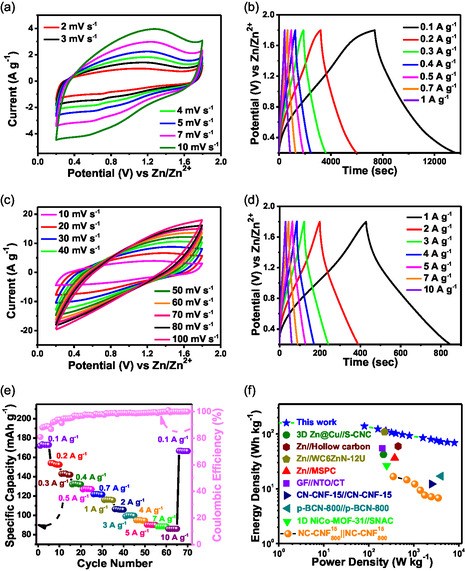
CV curves at scan rates of a) 2–10 mV s^−1^ and c) 10–100 mV s^−1^. GCD plots at current densities b) 0.1–1 A g^−1^ and d) 1–10 A g^−1^. e) Rate performance plot with Coulombic efficiency. f) Ragone plot of Zn||NC‐CNF^15^_800_ versus NC‐CNF^15^_800_||NC‐CNF^15^_800_ and other reported literature.

In order to construct a device with higher PD and ED than that of the symmetric SC, with an aim toward approaching a more practically applicable solution, a ZIHSC device (Zn||NC‐CNF^15^_800_) was prepared using metallic Zn plate as anode, NC‐CNF^15^_800_ coated Ti foil as cathode, and 2 M ZnSO_4_ as electrolyte. All the electrochemical experiments were performed within 0.2–1.8 V versus Zn/Zn^2+^ since there were no water‐splitting peaks observed within this range. The CV curves of Zn||NC‐CNF^15^_800_ ZIHSC at low (2–10 mV s^−1^) and high (10–100 mV s^−1^) sweep speeds are displayed in **Figure** **7**a,c, respectively. The curves show redox peaks, which are indicative of the anodic reaction carried out by the Faradic process involving Zn^2+^ ion deposition and stripping:^[^ ^52^, ^53^, ^54^, ^55^ ^]^

(5) $$\text{Zn }↔{\text{ Zn}}^{2+}+2e^{-}$$

Zn^2+^ cations are removed from the Zn anode during discharging and are drawn toward the NC‐CNF^15^_800_ cathode by electrostatic attraction. In the charging process, SO_4_^2−^ ions travel toward the NC‐CNF^15^_800_ electrode and Zn^2+^ ions migrate back to the Zn electrode. The GCD curves of Zn||NC‐CNF^15^_800_ at low (0.1–1 A g^−1^) and high (1–10 A g^−1^) current densities are illustrated in Figure 7b,d, respectively. The device reached a peak specific capacity of 173.5 mAh g^−1^ under 0.1 A g^−1^. As the current density rises from 0.1, 0.2, 0.3, 0.4, 0.5, 0.7, 1, 2, 3, 4, 5, 7 up to 10 A g^−1^ the specific capacity decreases in the order of 173.5, 154.1, 143.2, 132.8, 127.6, 122, 116.3, 106.4, 100, 95, 90.3, 88.5, 86.1 mAh g^−1^ respectively. The plot of rate capability of Zn||NC‐CNF^15^_800_ ZIHSC from 0.1 to 10 A g^−1^ is shown in Figure 7e. It demonstrates that although the capacity declines with increasing current density, it returns to 96.1% of its starting capacity when 0.1 A g^−1^ is once again applied. The maximum PD and ED values of the ZIHSC (determined using the Equation (S5) and (S6) respectively) were calculated to be 7998.9 W kg^−1^ and 138.8 Wh kg^−1^ respectively. Figure 7f shows the Ragone plot of ED versus PD, where the results are superior than those of the ZIHSCs and supercapacitors that have been reported recently, such as 3D Zn@Cu//S‐CNC,^[^ ^56^ ^]^ Zn//hollow carbon,^[^ ^57^ ^]^ Zn//WC6ZnN‐12U,^[^ ^58^ ^]^ Zn//MSPC,^[^ ^59^ ^]^ GF//NTO/CT,^[^ ^60^ ^]^ CN‐CNF‐15//CN‐CNF‐15,^[^ ^61^ ^]^ p‐BCN‐800//p‐BCN‐800,^[^ ^62^ ^]^ and 1D NiCo‐MOF‐31//SNAC.^[^ ^63^ ^]^ The excellent ED and PD values can be attributed to the well‐developed pore network and the N, O doping in the porous CNFs. The PD and ED values of the symmetric NC‐CNF^15^_800_||NC‐CNF^15^_800_ device have also been plotted in the same graph to get a clear comparison of how superior is the ZIHSC than the SC device. Apart from the redox humps appearing in the CV curves of the Zn||NC‐CNF^15^_800_ device, the curves also show approximately rectangular shape suggesting that the charge storage is both EDLC (capacitive contribution) and pseudocapacitive (diffusive contribution) in nature. A comparison of the electrochemical performance of Zn||NC‐CNF^15^_800_ versus reported porous carbon materials for ZIHSC cathodes is shown in **Table** **3**.

**Table 3:** Electrochemical performance of Zn||NC‐CNF^15^_800_ versus reported porous carbon materials for ZIHSC cathodes.

Material	Specific Capacity [mAh g^−1^]	Electrolyte	References
AC||Zn	121 (0.1 A g^−1^)	2 M ZnSO_4_	[65]
B, N‐codoped carbon||Zn	190.2 (0.1 A g^−1^)	3 M Zn (CF_3_SO_3_)_2_	[66]
O and N atoms‐doped carbon||Zn	141 (0.1 A g^−1^)	2 M ZnSO_4_	[67]
MCHS||Zn	174.7 (0.1 A g^−1^)	2 M ZnSO_4_	[68]
Poplar wood‐based derived carbon||Zn	111 (0.1 A g^−1^)	2 M ZnSO_4_	[58]
SN‐PCNT||Zn	152.6 (0.1 A g‐1)	2 M ZnSO_4_	[69]
PC800||Zn	179.8 (0.1 A g^−1^)	3 M Zn(ClO_4_)_2_	[70]
HPC‐600||Zn	169.4 (0.1 A g^−1^)	1 M Zn(CF_3_SO_3_)_2_	[26]
OPCNF‐20||Zn	136.4 (0.1 A g^−1^)	1 M ZnSO_4_	[16]
Zn||NC‐CNF^15^_800_	**173.5 (0.1 A g** ^**−1**^)	**2 M ZnSO**_**4**_	**This work**

To inspect diffusion and capacitive control on the charge storage process, the power law is employed.

(6) $$i\left(V\right)=av^{b}$$

**Figure 8:**
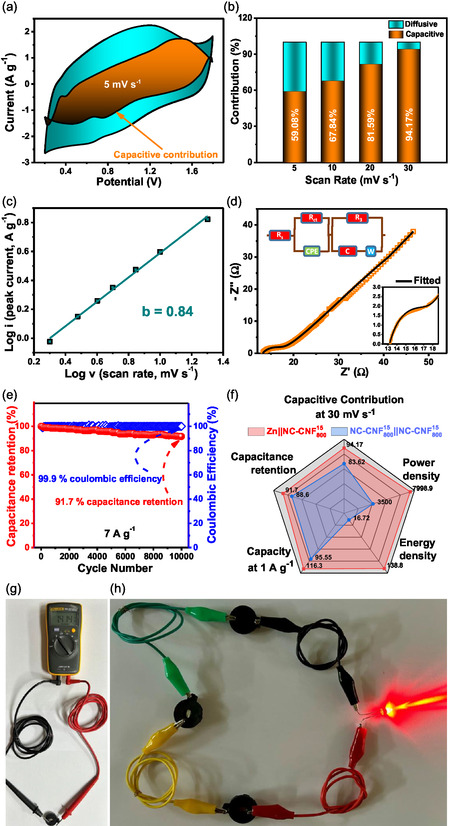
a) Capacitive contribution plot at 5 mV s^−1^. b) Capacitive and diffusive charge storage processes versus scan rates (5–30 mV s^−1^). c) log (*i*) versus log (*v*) profile. d) Nyquist plot and e) percent capacitance retention and Coulombic efficiency versus number of cycles at 7 A g^−1^ for Zn||NC‐CNF^15^_800_. f) Comparison of electrochemical performance of NC‐CNF^15^_800_||NC‐CNF^15^_800_ versus Zn||NC‐CNF^15^_800_. g) OCP measurement of an assembled ZIHSC device (Zn||NC‐CNF^15^_800_). h) Illumination of a 3 V red LED bulb by a series combination of three Zn||NC‐CNF^15^_800_ devices.

Here, the variable parameters are *a* and *b*, the current is indicated by *i*, and the sweep rate is indicated by *v*. The *b* value obtained from the slope of log (*i*) versus log (*v*) plot indicates the dominant charge storage mechanism. Generally, *b* **≈**0.5 suggests a diffusion‐controlled mechanism, while *b* **≈**1 indicates surface capacitive control. As shown in **Figure** **8**c, the log (*i*) versus log (*v*) plot reveals a *b* value of 0.84, indicating that both diffusive and capacitive pathways control the charge storage process. The percentage contributions of diffusion and capacitive‐controlled processes were calculated using Dunn's method. Figure 8a represents the capacitive contribution plot at 5 mV s^−1^, and the bar plot of capacitive versus diffusive control with increasing scan speeds is displayed in Figure 8b. The capacitive contributions were determined as 59.08, 67.84, 81.59, and 94.17% for scan speeds of 5, 10, 20, and 30 mV s^−1^, respectively. These results imply dominating EDLC behavior of the material since the capacitive contribution increases with increasing scan speeds. The electrochemical kinetics is clearly controlled by capacitive behavior, since it has more than half contribution in the charge storage process.^[^ ^64^ ^]^ *R*_ct_ and *R*_s_ values were determined as 3.69 and 13.17 Ω, respectively, from the EIS curve in Figure 8d, indicating effective transport of ions through the material. Also, to prove that electrolyte diffusion increases with increase in the amount of surface functional groups which depends on the optimum amount of NC doping, we performed galvanostatic intermittent titration technique (GITT) on Zn||NC‐CNF^10^_800_, Zn||NC‐CNF^15^_800_, and Zn||NC‐CNF^30^_800_ devices (Figure S17, Supporting Information). During both discharging and charging processes, it was found that the Zn^2+^‐ion diffusion coefficient ($D_{{\text{Zn}}^{2+}}$) values increased from NC‐CNF^10^_800_ to NC‐CNF^15^_800_ and then again decreased in NC‐CNF^30^_800_ in accordance with the fact that beyond 0.15 g of NC doping, NC incorporation in the nanofibers gets limited, and hence the amount of hydrophilic surface functional groups also decreases. The $D_{{\text{Zn}}^{2+}}$ values, calculated using Equation (S7), are mentioned in **Table** **4**. As shown in Figure 8e, the device also retained 91.7% of its capacity at 7 A g^−1^ beyond 10 k cycles. Finally, the overall charge storage performance of Zn||NC‐CNF^15^_800_ ZIHSC has been compared with that of NC‐CNF^15^_800_||NC‐CNF^15^_800_ SC in Figure 8f. It has been observed that the maximum PD (7998.9 W kg^−1^) and ED (138.8 Wh kg^−1^) values exhibited by the ZIHSC device were far greater than those of the SC (16.72 Wh kg^−1^ and 3500.1 W kg^−1^, respectively). Besides, the ZIHSC also exhibits greater capacity at 1 A g^−1^, higher retention of capacitance over 10 k cycles, and better capacitive contribution at 30 mV s^−1^, making it a better electrochemical charge storage device than that of the symmetric SC. The practical applicability of the prepared ZIHSC device was further studied by utilizing it to power up a 3 V red light emitting diode (LED) bulb. For this, the Zn||NC‐CNF^15^_800_ device was charged up to 1.8 V and allowed to rest for 4 h. After that, the open‐circuit potential (OCP) stabilized at ≈1.4 V (Figure 8g). In order to light up a 3 V red LED bulb, three of such devices were connected in series and the system was able to deliver sufficient power to illuminate the bulb for ≈60 s at maximum intensity (Figure 8h).

**Table 4:** Zn^2+^ ion diffusion coefficients ($D_{{\text{Zn}}^{2+}}$ ) in Zn||NC‐CNF^10^_800_, Zn||NC‐CNF^15^_800_, and Zn||NC‐CNF^30^_800_ setups from GITT analysis.

Sample Name	Zn^2+^ ion diffusion coefficient ($D_{{\text{Zn}}^{2+}}$)	
	Discharging [cm^2^ s^−1^]	Charging [cm^2^ s^−1^]
NC‐CNF^10^_800_	1.57 × 10^−9^–9.57 × 10^−9^	1.19 × 10^−12^–7.68 × 10^−9^
**NC‐CNF** ^**15**^_**800**_	**1.85 × 10** ^**−9**^ **–1.28 × 10** ^**−8**^	**6.07 × 10** ^**−10**^ **–8.89 × 10** ^**−9**^
NC‐CNF^30^_800_	1.83 × 10^−9^–1.27 × 10^−8^	3.21 × 10^−10^–8.54 × 10^−9^

## 5 Conclusion

In the present work, NC‐incorporated AC nanofibers (NC‐CNFs) with varying NC loadings were synthesized by electrospinning followed by stabilization, carbonization, and finally KOH activation at different temperatures. The optimized nanofibers were found to have a well‐developed porous structure with an optimum mix of micro‐ and mesopores. The NC‐CNFs were initially tested in a three‐electrode setup where the optimized sample showed an excellent C_s_ of 418 F g^−1^ at 1 A g^−1^ in 1 M H_2_SO_4_. It was then utilized to prepare a symmetric SC device which shows a C_s_ of 245.71 F g^−1^ at 1 A g^−1^ along with maximum PD and ED values of 16.72 Wh kg^−1^ and 3500.1 W kg^−1^ respectively. Most importantly, the NC‐CNFs exhibited an exceptional performance as ZIHSC cathode with the highest specific capacity value at 0.1 A g^−1^ being 173.5 mAh g^−1^ in 2 M ZnSO_4_. The porosity of the nanofibers helps to enhance the cyclability of the electrode by facilitating effective electrolyte ion transfer. Excellent ED (up to 138.8 Wh kg^−1^) and PD (up to 7998.9 W kg^−1^) values were displayed by the ZIHSC device, demonstrating 91.7% cyclic stability in 2 M ZnSO_4_ over 10 k cycles. The superior charge storage capability of the NC‐CNFs is ascribed to the N, O functional groups on the nanofiber surface, elevated surface area, and their porous nanofiber structure. This study highlights the substantial enhancement in charge storage performance of ZIHSC achieved by introducing NC in activated electrospun PAN‐derived CNFs.

## Conflict of Interest

The authors declare no conflict of interest.

## Author Contributions

**Sayak Roy**: writing—review & editing, data curation. **Rajib Samanta**: writing—review & editing, data curation. **Sudip Barman**: conceptualization, supervision,writing—review & editing.

## Supporting information

Supplementary Material

## Data Availability

Data supporting the findings of this study will be made available from the corresponding author upon reasonable request.
