# Supplementary material for: Electrospun N‐Doped Carbon–Carbon Nanofibers with Enhanced Porosity for High‐Performance Zinc‐Ion Hybrid Supercapacitor Application
Source: Small Sci. 2025 Feb 25;5(4):2400426. doi: 10.1002/smsc.202400426 (PMC12244998; doi:10.1002/smsc.202400426)
Supplement: Supplementary file 1 — Supplementary Material [file SMSC-5-2400426-s001.pdf]

Supporting Information

Electrospun N-Doped Carbon-Carbon Nanofibers with Enhanced Porosity for High Performance Zinc Ion Hybrid Supercapacitor Application

Sayak Roy [a,b,c], Rajib Samanta [a,b,c], Sudip Barman[a,b,c]*

[a]School of Chemical Sciences, National Institute of Science Education and Research (NISER), Bhubaneswar, Orissa 752050, India

[b]Homi Bhabha National Institute (HBNI), 2nd floor, BARC Training School Complex, Anushakti Nagar, Mumbai, Maharashtra 400094, India

[c]Centre for Interdisciplinary Sciences, National Institute of Science Education and Research (NISER), Bhubaneswar, Orissa 752050, India

E-mail address: sbarman@niser.ac.in.

Tel.: +91 6742494183

Materials

Formamide ($HCONH_2$), N,N-Dimethylformamide (DMF), and Glass micro-fiber (GF-C) separator was purchased from Merck, India. Polyacrylonitrile (PAN) was purchased from Sigma-Aldrich. Potassium Hydroxide (KOH), Hydrochloric acid (HCl), and Sulphuric acid (H_2SO_4) were purchased from CDH chemicals, India. PVDF binder was purchased from MTI corporation. Deionized H_2O was used to carry out all experiments.

Material Characterization

The powder x-ray diffraction patterns (p-XRD) of samples were measured by Bruker X-ray diffractometer (DAVINCI D8 ADVANCE equipped with Cu Kα source of wavelength 0.154 nm). The morphological characterization was investigated by a Field-emission scanning electron microscope (FESEM) system (Model-∑igma, Carl Zeiss, Germany) and Transmission Electron Microscopy (TEM) instrument operated at 200 kV (F200, JEOL). X-ray Photoelectron Spectroscopy (XPS) measurement was done using AXIS ULTRA (Kratos) instrument where a monochromated Al-kα source was used. XPS was taken from the sample deposited on the silicon wafer. The N_2 physisorption isotherm was collected using AUTOSORB-1 (Quantachrome). The pore size distribution (PSD) was calculated using the nonlocal density functional theory (NLDFT) model while the micropore analysis was carried out using the t-plot method. LabRAM HR Evolution, Horiba Scientific, and Raman Spectrometer were used for Raman analysis using a 532 nm laser source. Electrochemical measurements were performed using CS310 Electrochemical Workstation (Corrtest Instruments).

Electrochemical Characterization

Fabrication of electrodes was carried out by mixing PVDF binder, conductive carbon, and NC-CNF sample with a mass ratio of 10:10:80 followed by coating over Titanium foil (1*1 cm^2). The electrodes were dried at 85℃ in vacuum. The mass loading of each electrode was close to 1 mg. The single electrode performance was measured in an electrolyte of 1 M H_2SO_4 in a three-electrode system configuration with Ag/AgCl as reference and platinum mesh as counter electrode while the symmetric supercapacitor (SC) device and asymmetric zinc ion hybrid supercapacitor (ZIHSC) device were tested in a two-electrode system. The devices were prepared using glass microfibre filter paper as a separator in a CR2032 coin cell configuration. From a single electrode, the specific capacitance (C_s) calculation was carried out from Galvanostatic Charge discharge (GCD) curves using the following equation

$$C_s = \frac{I\Delta \mathrm{t}}{\mathrm{m}(V_f - V_i)} \quad \text{(S1)}$$

Where,

I = current applied (A)

Δt = discharge time (sec)

m = mass of the active material (g)

$V_f - V_i$ = Voltage window (V)

For a symmetric SC device, the calculation was done using the following equations.

$$C_s = \frac{2I\Delta t}{m(V_f - V_i)} \quad (S2)$$

$$ED_{SC} = \frac{C_{cell}\Delta V^2}{2\times3.6} = \frac{C_S\Delta V^2}{8\times3.6} \quad (S3)$$

$$PD_{SC} = \frac{ED\times3600}{t} \quad (S4)$$

Where,

ED = Energy density, and PD = power density.

The energy density (E, Wh kg^{-1}) and power density (P, W kg^{-1}) of the ZIHSC are calculated based on the following equations, respectively.

$$ED_{ZIHSC} = 0.5 \times C\Delta V \quad (S5)$$

$$PD_{ZIHSC} = \frac{ED\times3600}{t} \quad (S6)$$

The Zn^{2+} ion diffusion coefficients ($D_{Zn^{2+}}$) are calculated for the ZIHSC device using the following equation.

$$D_{Zn^{2+}} = \left(\frac{4L^2}{\pi\tau}\right) \times \left(\frac{\Delta E_S}{\Delta E_\tau}\right)^2 \quad (S7)$$

Where,

L = effective thickness of the electrode coating, τ = pulse time, ΔE_S = open circuit potential (OCP) between two adjacent pulses, and ΔE_τ = potential change caused by an impulse

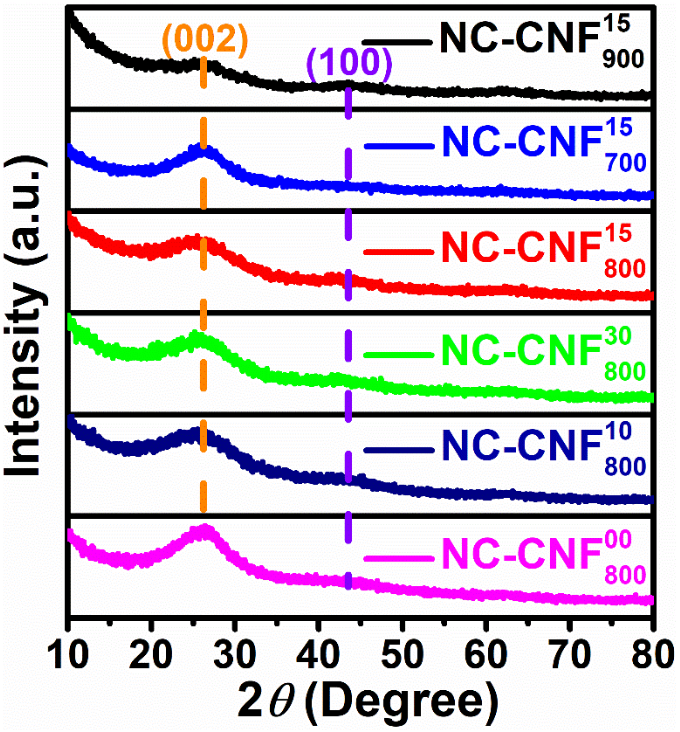

Figure S1 p-XRD patterns of NC-CNFs.

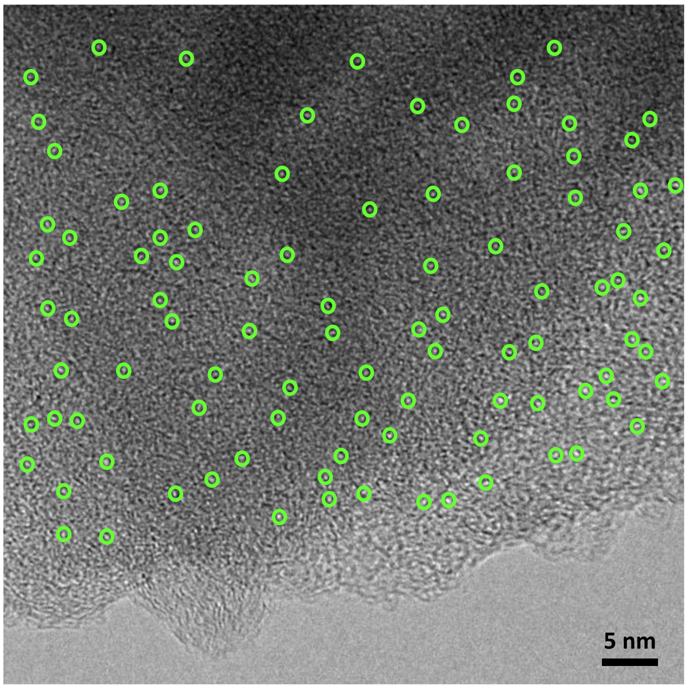

Figure S2 HRTEM image of NC-CNF$^{15}_{800}$ with marked micropores.

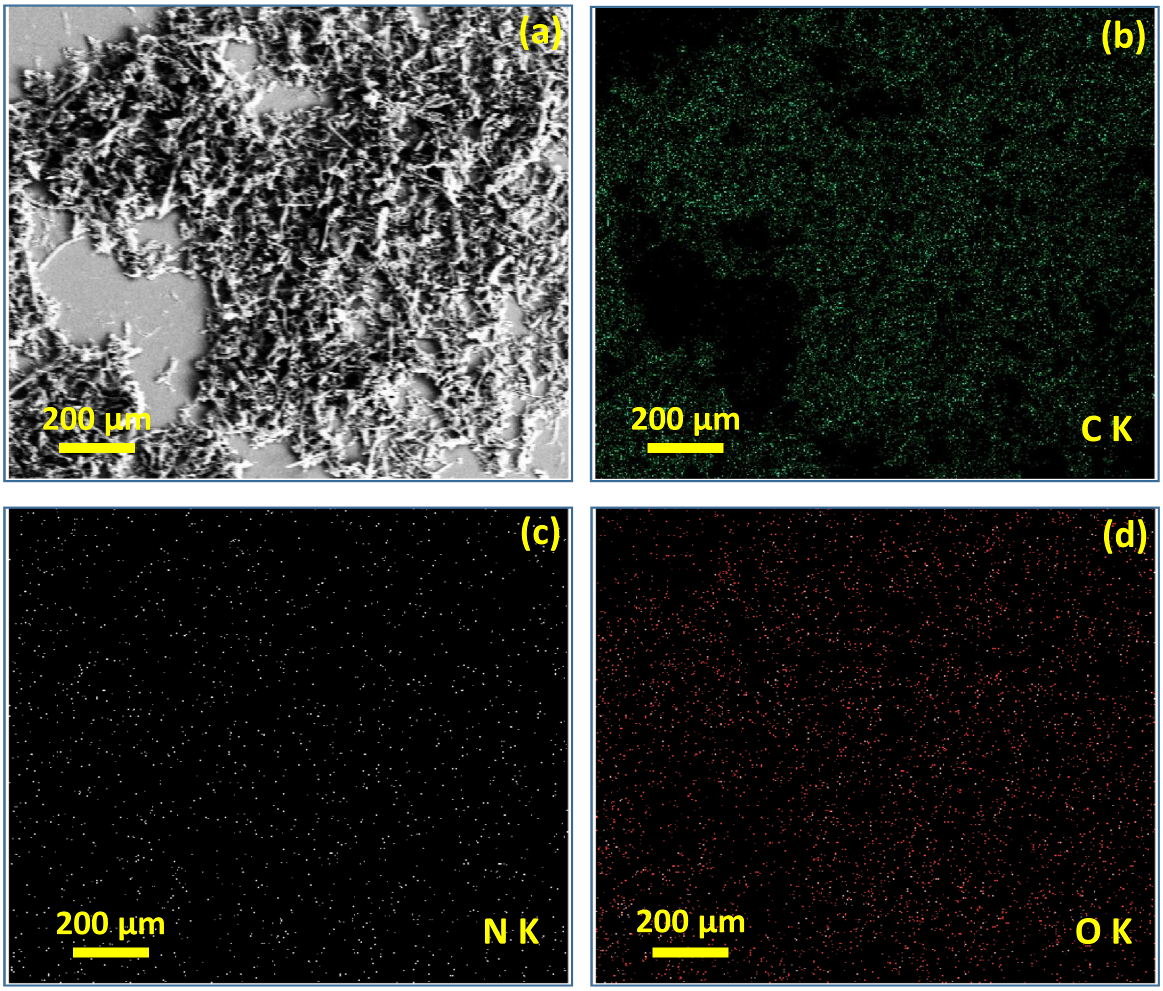

Figure S3 (a) Field of view image of elemental mapping from SEM EDX, distribution of (b) C, (c) N, and (d) O in the sample NC-CNF$^{15}_{800}$.

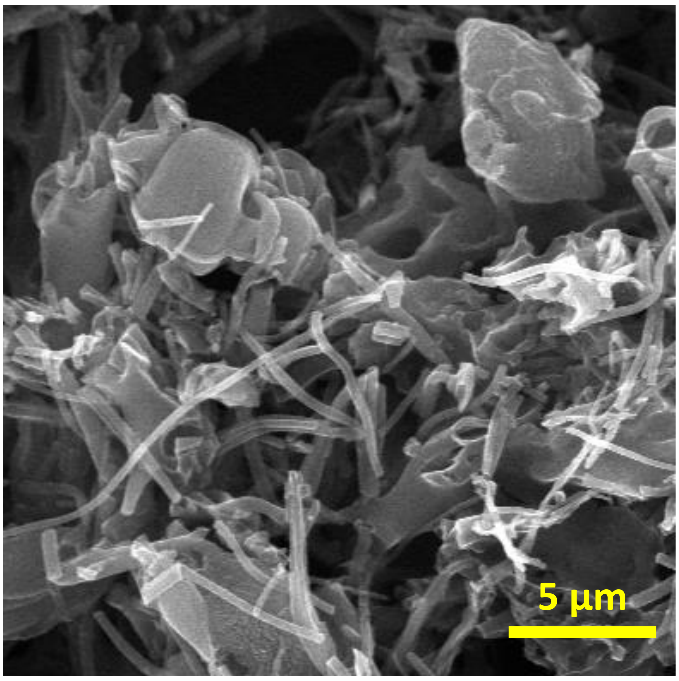

Figure S4 FESEM image of NC-CNF$^{30}_{800}$ showing nanofibers along with unincorporated NC.

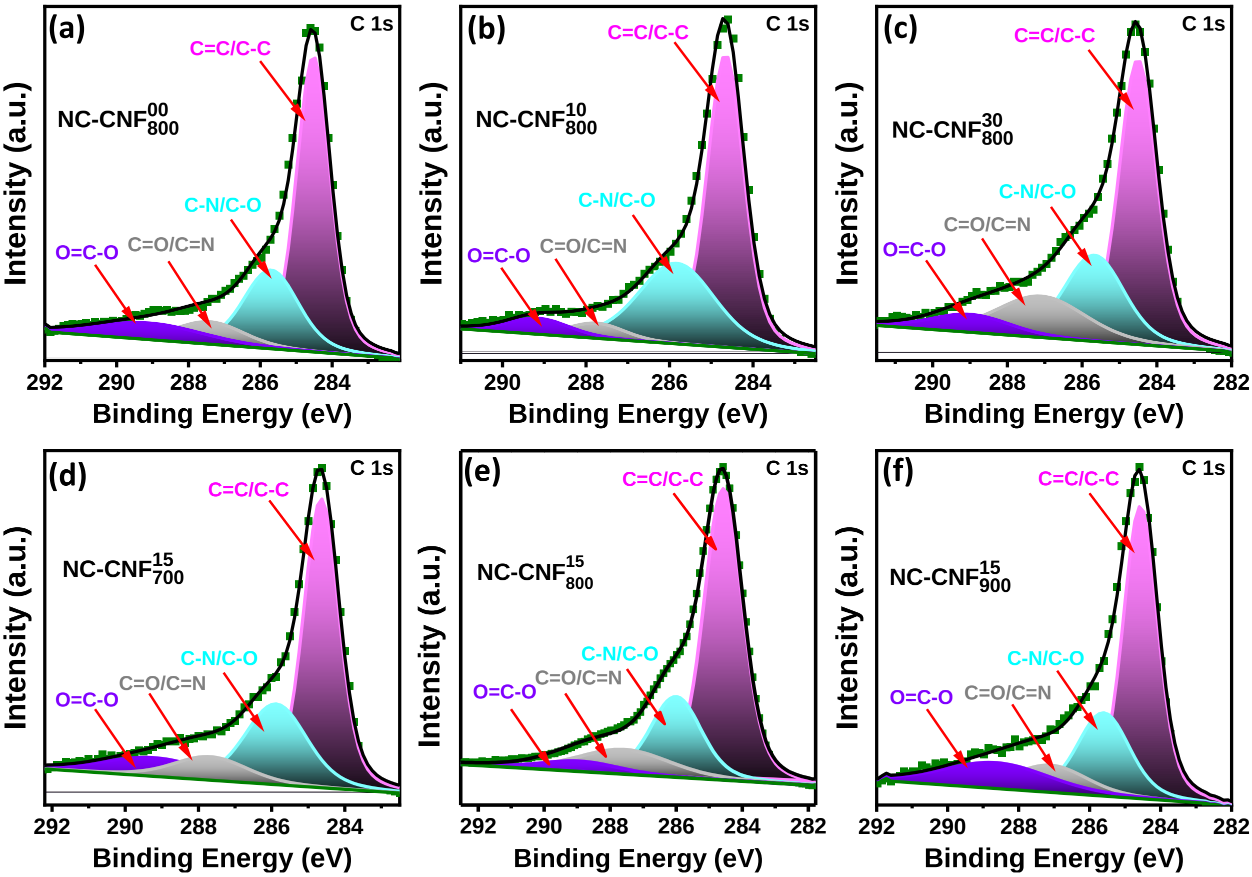

Figure S5 Deconvoluted C 1s XPS spectra of NC-CNF$^{x}_{y}$ samples.

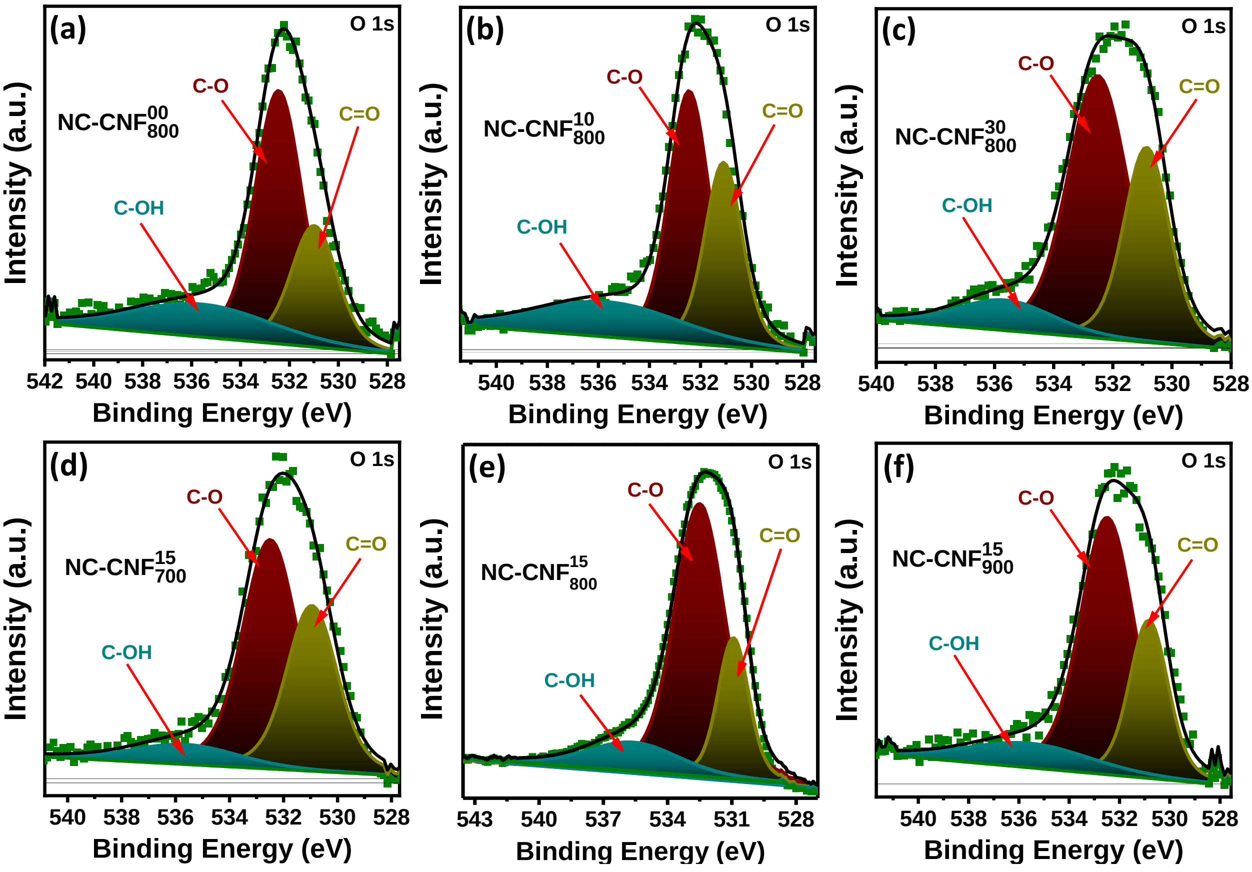

Figure S6 Deconvoluted O 1s XPS spectra of NC-CNF$^x{}_y$ samples.

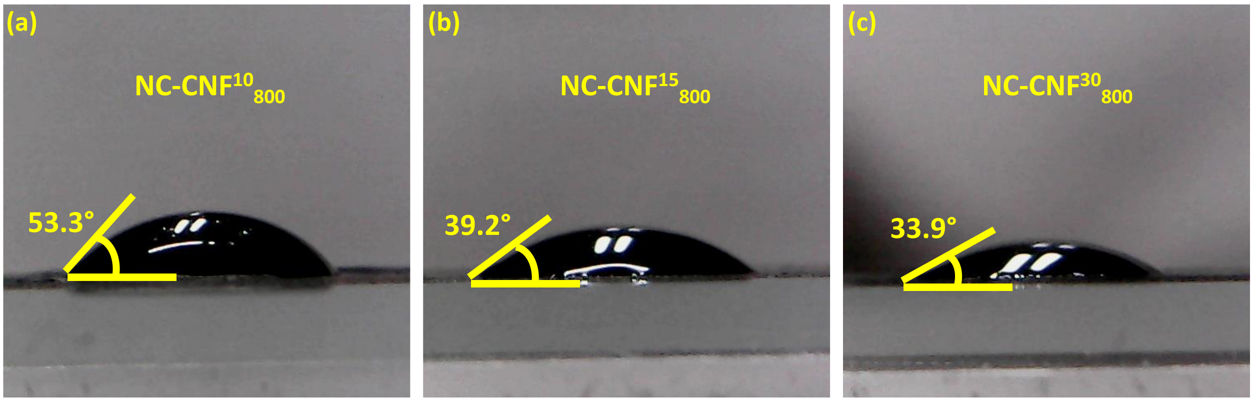

Figure S7 Contact angles of a water droplet on (a) NC-CNF$^{10}{}_{800}$, (b) NC-CNF$^{15}{}_{800}$, and (c) NC-CNF$^{30}{}_{800}$ samples

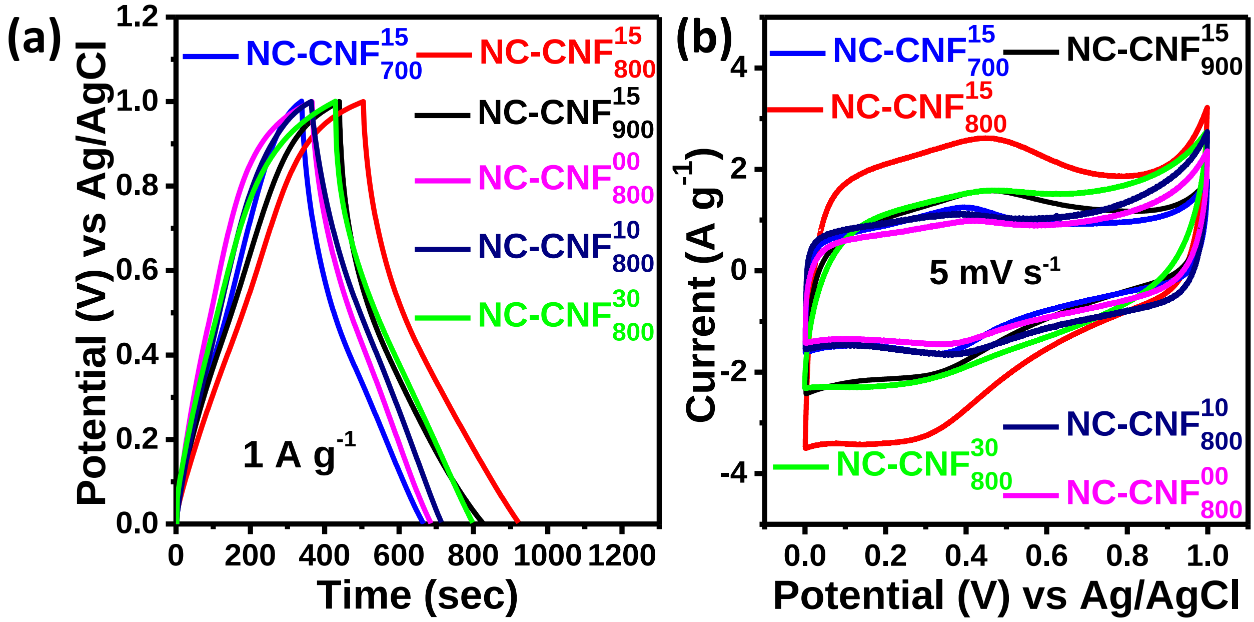

Figure S8 GCD and CV profiles of NC-CNF$^{x}_{y}$ samples.

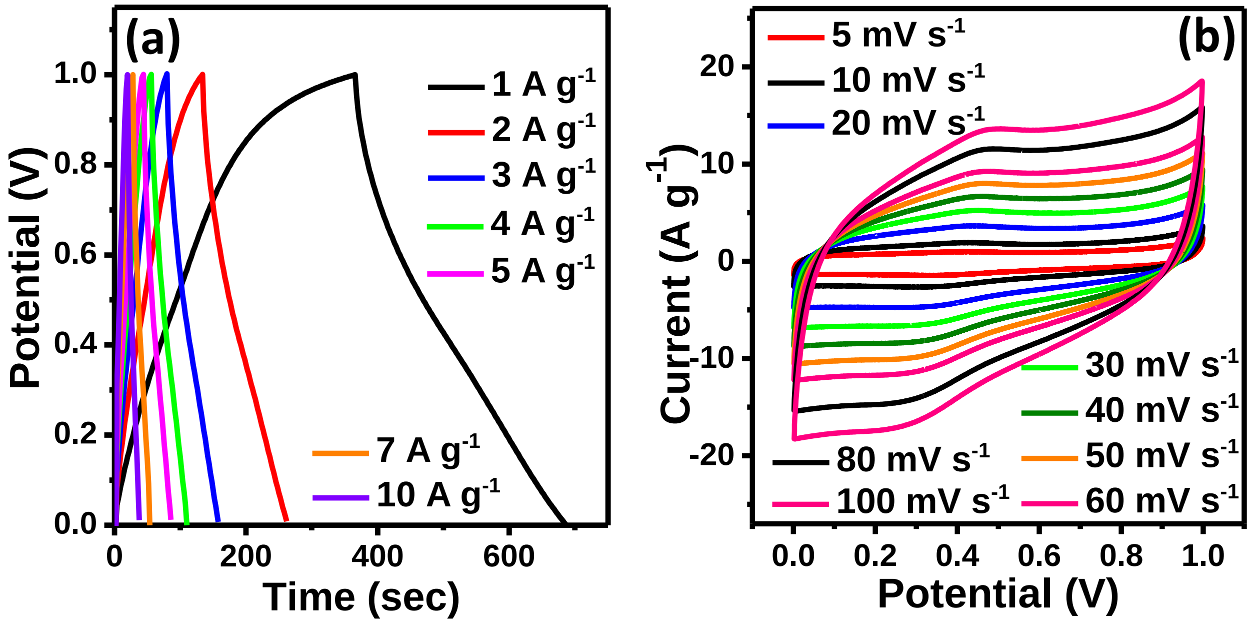

Figure S9 GCD and CV profiles of NC-CNF$^{00}_{800}$.

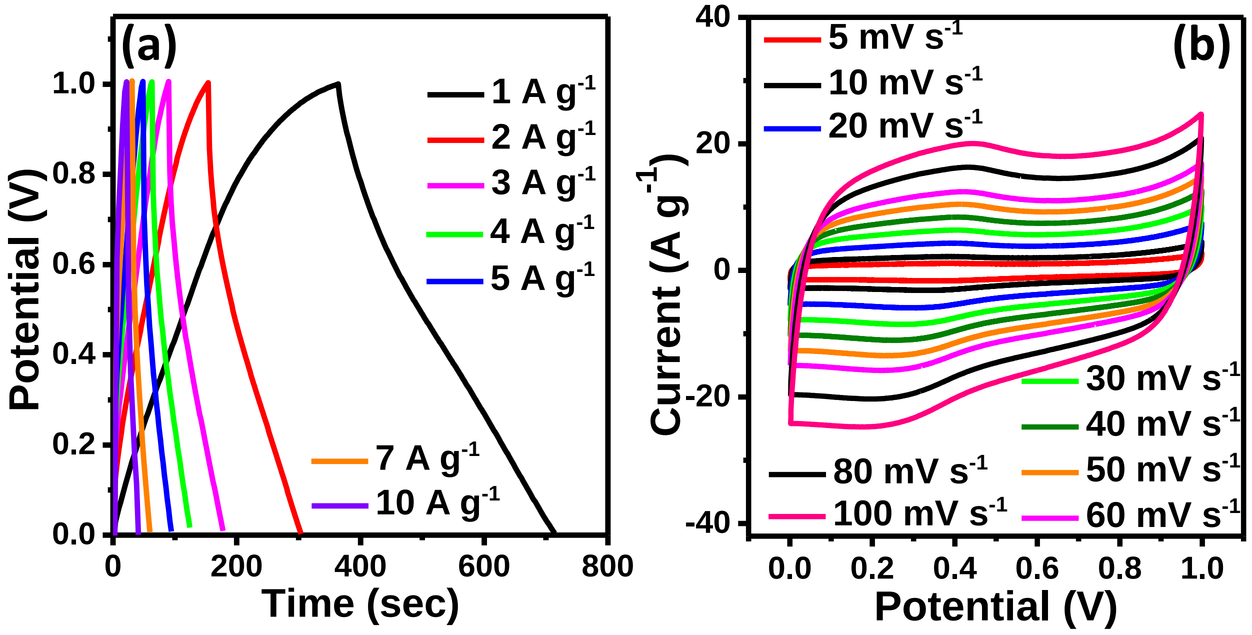

Figure S10 GCD and CV profiles of NC-CNF$^{10}{}_{800}$.

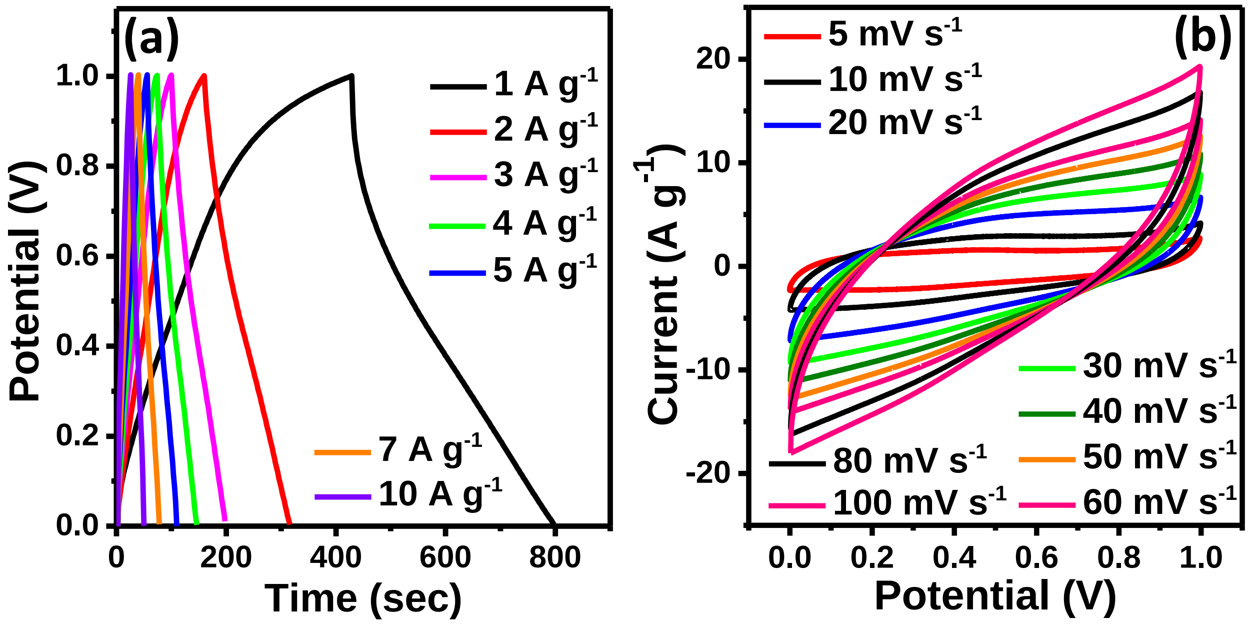

Figure S11 GCD and CV profiles of NC-CNF$^{30}{}_{800}$.

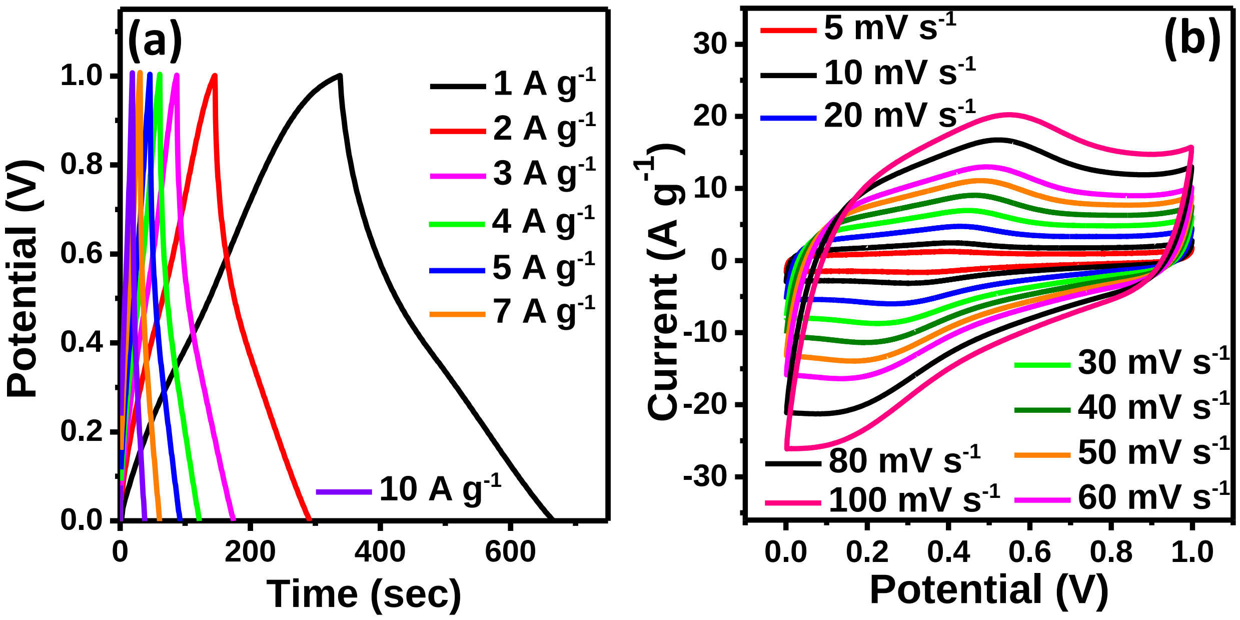

Figure S12 GCD and CV profiles of NC-CNF$^{15}_{700}$.

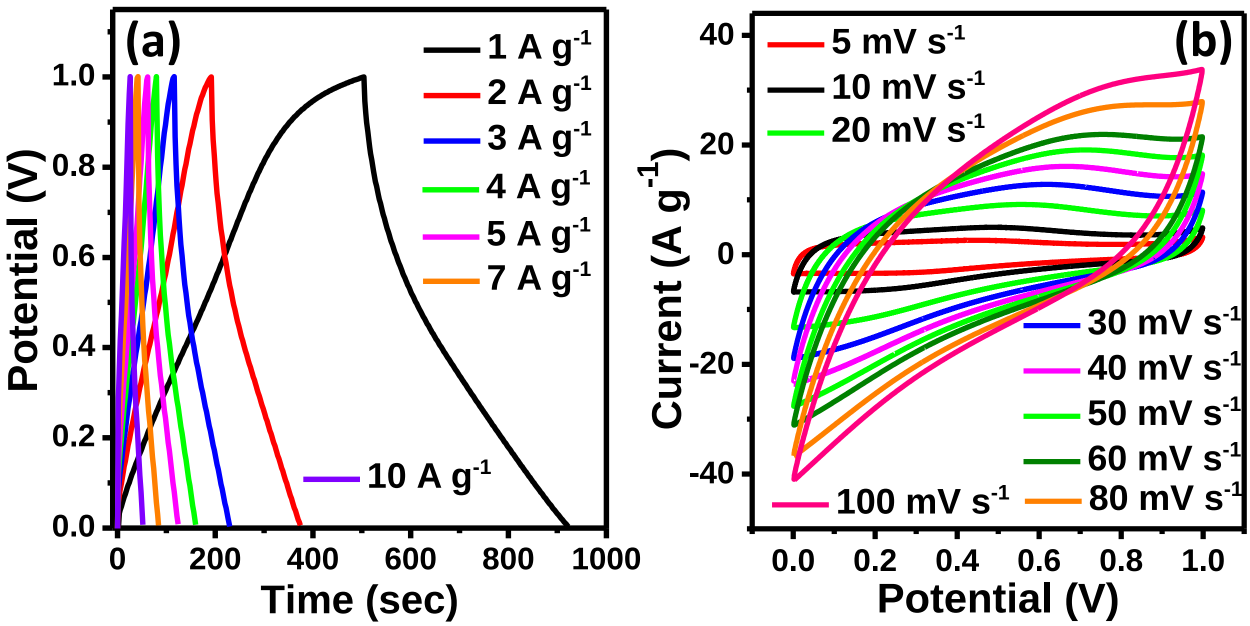

Figure S13 GCD and CV profiles of NC-CNF$^{15}_{800}$.

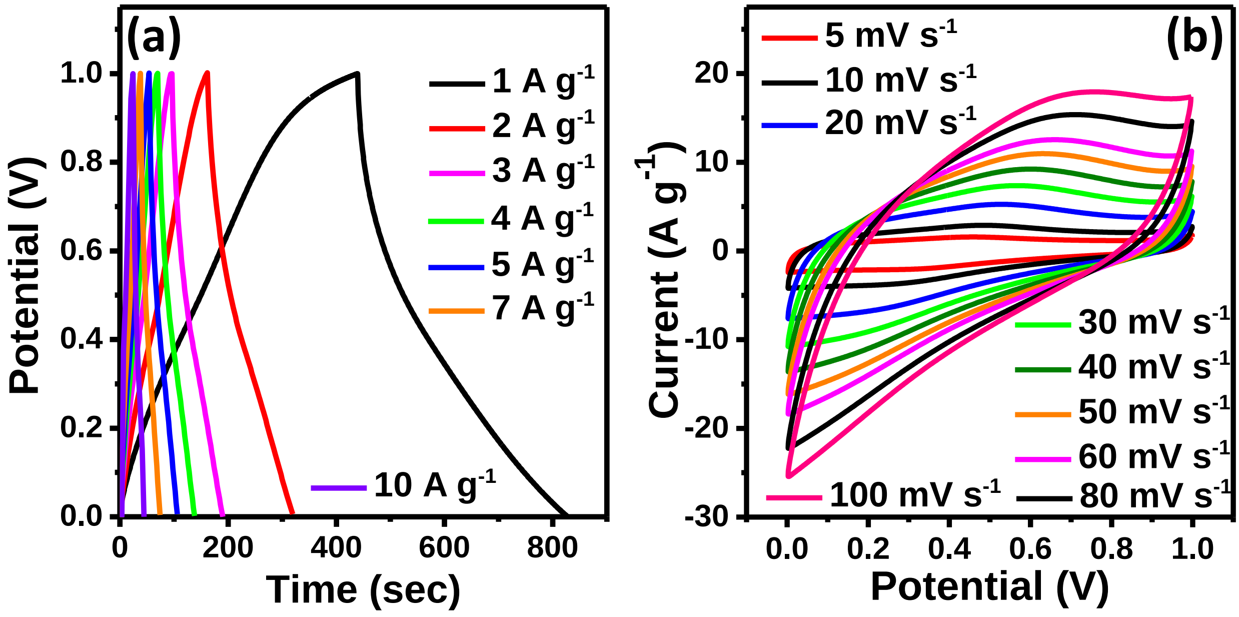

Figure S14 GCD and CV profiles of NC-CNF$^{15}_{900}$.

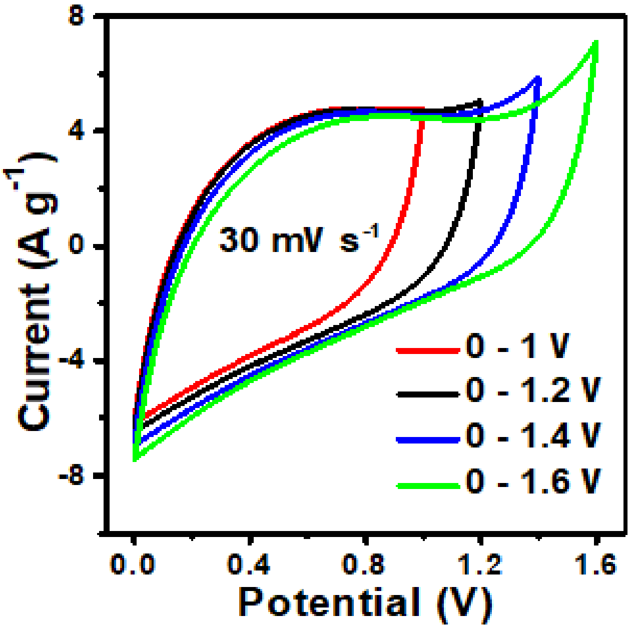

Figure S15 CV curves of NC-CNF$^{15}_{800}$||NC-CNF$^{15}_{800}$ device at the different potential ranges from 0 to 1 V up to 1.6 V in 1 M H_2SO_4.

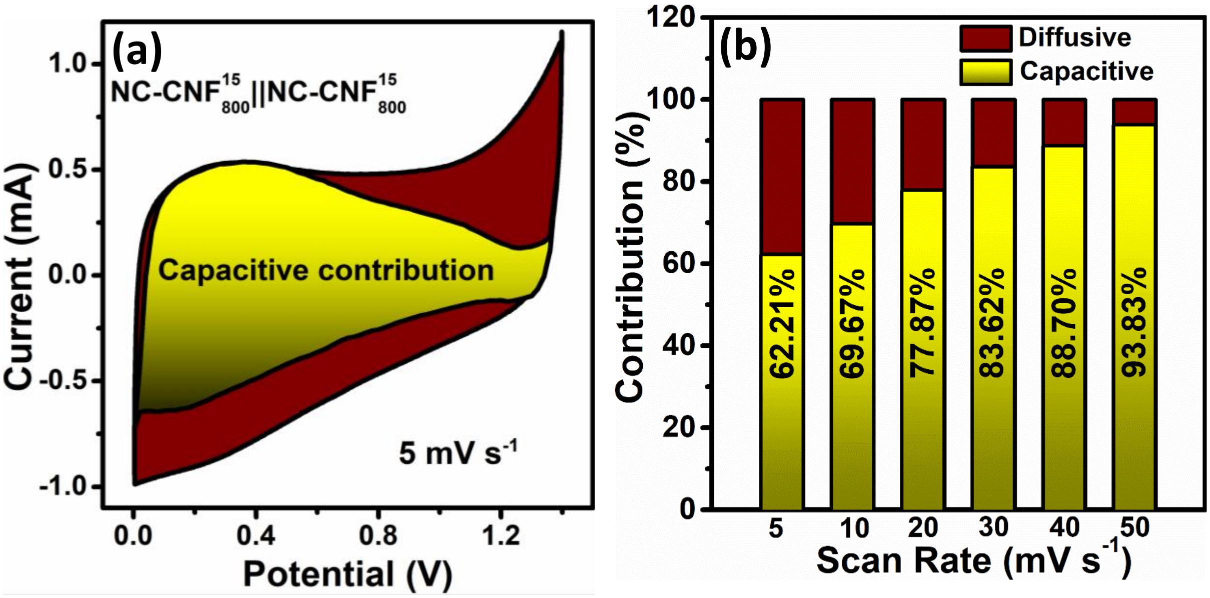

Figure S16 (a) Capacitive contribution plot at 5 mV s^{-1} (b) Capacitive and diffusive charge storage processes *vs.* scan rates (5-50 mV s^{-1}) of NC-CNF$^{15}_{800}$||NC-CNF$^{15}_{800}$ SC.

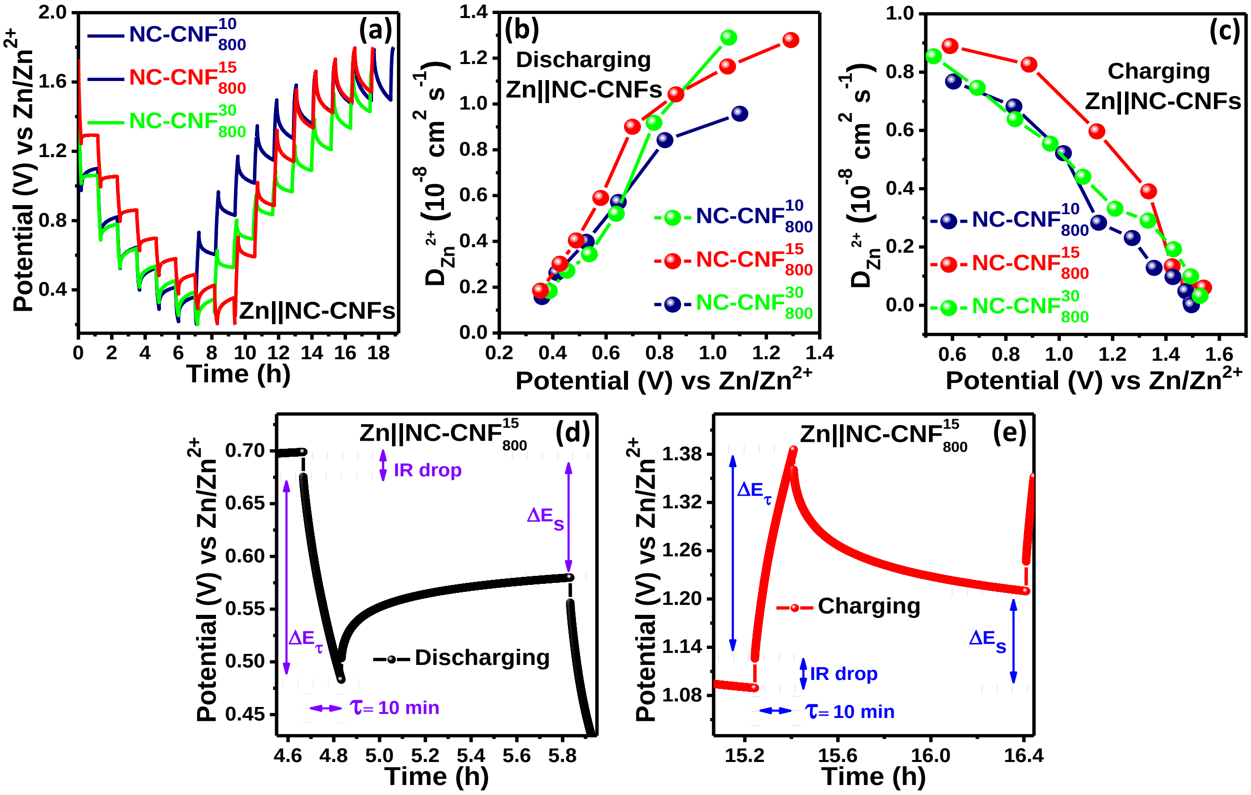

Figure S17 (a) GITT discharging and charging cycles of Zn||NC-CNFs, $D_{Zn^{2+}}$ values of Zn||NC-CNFs during (b) discharging and (c) charging processes, and (d, e) Necessary parameters to consider for $D_{Zn^{2+}}$ calculation during GITT discharging and charging respectively.

Table S1. Mass % of C, N, and O present in NC-$CNF^x{}_y$ samples (a) with NC doping variation (b) with activation temperature variation, as calculated from SEM EDX analysis.

Sample Name	C (%)	N (%)	O (%)
	**Table (a)**		
NC-$CNF^{10}{}_{800}$	75.30	5.40	19.30
**NC-$CNF^{15}{}_{800}$**	**73.30**	**7.60**	**20.10**
NC-$CNF^{30}{}_{800}$	67.58	10.60	21.82
	**Table (b)**		
NC-$CNF^{15}{}_{700}$	70.30	11.60	18.10
**NC-$CNF^{15}{}_{800}$**	**73.30**	**7.60**	**20.10**
NC-$CNF^{15}{}_{900}$	71.69	6.00	22.31

Table S2. R_s and R_{ct} values of NC-CNF samples from Nyquist plots in 1 M H_2SO_4 solution.

Sample Name	R_s	R_{ct}
NC-$CNF^{00}{}_{800}$	1.38	5.10
NC-$CNF^{10}{}_{800}$	1.31	2.06
NC-$CNF^{30}{}_{800}$	1.28	1.86
NC-$CNF^{15}{}_{800}$	1.23	0.23
NC-$CNF^{15}{}_{700}$	1.31	3.94
NC-$CNF^{15}{}_{900}$	1.25	0.91
